# Supplementary material for: Calibration of individual-based models to epidemiological data: A systematic review
Source: PLoS Comput Biol. 2020 May 11;16(5):e1007893. doi: 10.1371/journal.pcbi.1007893 (PMC7241852; doi:10.1371/journal.pcbi.1007893)
Supplement: S1 Table — (DOCX) [file pcbi.1007893.s001.docx]

**S1 Table. Articles included for review.**

| Author | Year | Journal | Pathogen | Parameter search strategy | Goal ^a^ |
| --- | --- | --- | --- | --- | --- |
| Abuelezam *et al*. [1] | 2016 | American Journal of Epidemiology | HIV | Sampling | I |
| Adams *et al*. [2] | 2018 | BMC Public Health | HIV | Unidentifiable | B |
| Bershteyn *et al*. [3] | 2013 | Journal of the Royal Society Interface | HIV | Optimization | I + B |
| Borre *et al*. [4] | 2017 | The Journal of Infectious Diseases | HIV | Unidentifiable | I |
| Brookmeyer *et al*. [5] | 2014 | PLOS ONE | HIV | Optimization | I |
| Cambiano *et al*. [6] | 2018 | Lancet Infectious Disease | HIV | Sampling | I |
| Cameron *et al*. [7] | 2015 | Nature Communications | Malaria | Sampling | I |
| Chang *et al*. [8] | 2018 | BMC Medicine | TB | Sampling | B |
| Chen *et al*. [9] | 2018 | Infectious Diseases of Poverty | Malaria | Unidentifiable | B |
| Ciaranello *et al*. [10] | 2013 | PLOS ONE | HIV | Sampling | O |
| DeVos *et al*. [11] | 2014 | AIDS | HIV | Unidentifiable | I |
| Eckhoff *et al*. [12] | 2013 | American Journal of Tropical Medicine and Hygiene | Malaria | Unidentifiable | I |
| Eckhoff *et al*. [13] | 2017 | PNAS Population Biology | Malaria | Unidentifiable | I |
| Escudero *et al*. [14] | 2016 | AIDS | HIV | Unidentifiable | O |
| Escudero *et al*. [15] | 2017 | BMC Public Health | HIV | Unidentifiable | O |
| Espindola *et al*. [16] | 2017 | Int Health | TB | Unidentifiable | I |
| Fu *et al*. [17] | 2016 | Mathematical Biosciences | HIV | Informal | B |
| Gantenberg *et al*. [18] | 2018 | PLOS ONE | HIV | Informal | I |
| Gerardin *et al*. [19] | 2015 | Malaria Journal | Malaria | Informal^b^ | I |
| Gerardin *et al*. [20] | 2015 | BMC Infectious Diseases | Malaria | Informal^b^ | I |
| Goedel *et al*. [21] | 2018 | Journal of Acquired Immune Deficiency Syndromes (1999) | HIV | Optimization | B |
| Gopalappa *et al*. [22] | 2016 | Medical Decision Making | HIV | Informal | O |
| Gopalappa *et al*. [23] | 2017 | AIDS | HIV | Informal | I |
| Gray *et al*. [24] | 2013 | PLOS ONE | HIV | Unidentifiable | I |
| Herbeck *et al*. [25] | 2016 | Virus Evolution | HIV | Unidentifiable | I |
| Hontelez *et al*. [26] | 2013 | PLOS Medicine | HIV | Sampling | I |
| Huynh *et al*. [27] | 2015 | BMC Medicine | TB | Sampling | I |
| Jewell *et al*. [28] | 2015 | PLOS ONE | HIV | Optimization | I |
| Johnson *et al*. [29] | 2016 | Sexually Transmitted Diseases | HIV | Sampling | O |
| Kasaie *et al*. [30] | 2014 | American Journal of Respiratory and Critical Care Medicine | TB | Unidentifiable | I |
| Kasaie *et al*. [31] | 2017 | Journal of Acquired Immune Deficiency Syndromes | HIV | Unidentifiable | I |
| Kasaie *et al*. [32] | 2018 | Sexually Transmitted Diseases | HIV | Optimization | I |
| Kessler *et al*. [33] | 2015 | Value in Health | HIV | Unidentifiable | I |
| Khademi *et al*. [34] | 2015 | Value in Health | HIV | Unidentifiable | O |
| Klein *et al*. [35] | 2015 | International Health | HIV | Optimization | I |
| Knight *et al*. [36] | 2015 | PLOS ONE | HIV, TB | Optimization | I |
| Knittel *et al*. [37] | 2015 | Social Science & Medicine | HIV | Informal | B |
| Luo *et al*. [38] | 2018 | JMIR Public Health and Surveillance | HIV | Optimization | I |
| Luz *et al*. [39] | 2015 | Journal of Acquired Immune Deficiency Syndromes (1999) | HIV | Unidentifiable | I |
| Marshall *et al*. [40] | 2018 | The Lancet. HIV | HIV | Optimization | I |
| McCarthy *et al*. [41] | 2015 | Malaria Journal | Malaria | Informal^b^ | I |
| McCormick *et al*. [42] | 2014 | PLOS ONE | HIV | Sampling | O |
| McCormick *et al*. [43] | 2017 | Epidemics | HIV | Sampling | I |
| McCreesh *et al*. [44] | 2017 | BMC Infectious Diseases | HIV | Sampling | I |
| McCreesh *et al*. [45] | 2017 | BMC Infectious disease | HIV | Sampling | I |
| McCreesh *et al*. [46] | 2018 | PLOS ONE | HIV | Sampling | I |
| Monteiro *et al*. [47] | 2015 | International Journal of Public Health | HIV | Unidentifiable | I |
| Nakagawa *et al*. [48] | 2015 | PLOS ONE | HIV | Unidentifiable | O |
| Nakagawa *et al*. [49] | 2016 | Epidemiology | HIV | Sampling | O |
| Nakagawa *et al*. [50] | 2017 | AIDS | HIV | Sampling | O |
| Nalugoda *et al*. [51] | 2016 | eLife | HIV | Unidentifiable | B |
| Nguyen *et al*. [52] | 2015 | The Lancet Global Health | Malaria | Unidentifiable | I |
| Nguyen *et al*. [53] | 2018 | Clinical Infectious Diseases | HIV | Unidentifiable | I |
| Olney *et al*. [54] | 2016 | Lancet HIV | HIV | Informal | I |
| Patel *et al*. [55] | 2017 | Medicine | HIV | Unidentifiable | I |
| Penny *et al*. [56] | 2015 | BMC Medicine | Malaria | Sampling | I |
| Penny *et al*. [57] | 2015 | Malaria Journal | Malaria | Sampling | O |
| Phillips *et al*. [58] | 2013 | PLOS ONE | HIV | Sampling | B |
| Phillips *et al*. [59] | 2015 | AIDS | HIV | Sampling | I |
| Phillips *et al*. [60] | 2016 | PLOS ONE | HIV | Unidentifiable | I |
| Pizzitutti *et al*. [61] | 2015 | Malaria Journal | Malaria | Sampling | I + O |
| Pizzitutti *et al*. [62] | 2018 | PLOS ONE | Malaria | Sampling | B |
| Prats *et al*. [63] | 2016 | Frontiers in Microbiology | TB | Unidentifiable | I + O |
| Rigby *et al*. [64] | 2017 | Infectious Disease Modelling | HIV | Unidentifiable | B |
| Romero-Severson *et al*. [65] | 2013 | Epidemiology | HIV | Optimization | B |
| Sauboin *et al*. [66] | 2015 | Malaria Journal | Malaria | Optimization | I |
| Schalkwyk *et al*. [67] | 2018 | Sexually Transmitted Infections | HIV | Sampling | B |
| Schneider *et al*. [68] | 2014 | Clinical Infectious Diseases | HIV | Unidentifiable | I |
| Selvaraj *et al*. [69] | 2018 | BMC Infectious Disease | Malaria | Informal^b^ | I + O |
| Sharma *et al*. [70] | 2018 | AIDS | HIV | Unidentifiable | I |
| Shcherbacheva *et al*. [71] | 2018 | Mathematical Biosciences | Malaria | Sampling | B |
| Shrestha *et al*. [72] | 2017 | American Journal of Respiratory and Critical Care Medicine | TB | Optimization | B |
| Shrestha *et al*. [73] | 2017 | American Journal of Epidemiology | HIV | Sampling | I |
| Smit *et al*. [74] | 2018 | AIDS | HIV | Unidentifiable | O |
| Smith *et al*. [75] | 2015 | Lancet HIV | HIV | Informal | I |
| Steen *et al*. [76] | 2014 | AIDS | HIV | Unidentifiable | I + B |
| Stevens *et al*. [77] | 2018 | PLOS ONE | HIV | Unidentifiable | I |
| Stevens *et al*. [78] | 2018 | Journal of Acquired Immune Deficiency Syndromes (JAIDS) | HIV | Unidentifiable | I |
| Suen *et al*. [79] | 2014 | PLOS ONE | TB | Optimization | I |
| Suen *et al*. [80] | 2015 | The International Journal of Tuberculosis and Lung Disease | TB | Optimization | I |
| Tuite *et al*. [81] | 2017 | BMC Public Health | TB | Sampling | I |
| Vandewalle *et al*. [82] | 2016 | PLOS ONE | HIV | Informal | B + O |
| Walensky *et al*. [83] | 2016 | Annals of Internal Medicine | HIV | Unidentifiable | O |
| White *et al*. [84] | 2018 | Nature Communications | Malaria | Sampling | I |

**^a^ Goals of the articles as depicted in the last column; I: Investigating the effect of an intervention on the infectious disease outbreak, B: Investigating a behavioral or biological explanation for the observed infectious disease outbreak, O: Other goals including estimation of parameters or model development.**

**^b^ Calibration involved a hybrid approach where both algorithmic (sampling from the prior + IMIS) and informal methods were used. As mentioned in the methods section we classified hybrid approaches as informal parameter search strategies.**

**References**

[1] Abuelezam NN, McCormick AW, Fussell T, Afriyie AN, Wood R, DeGruttola V, et al. Can the Heterosexual HIV Epidemic be Eliminated in South Africa Using Combination Prevention? A Modeling Analysis. American journal of epidemiology. 2016 aug;184(3):239–248.

[2] Adams JW, Lurie MN, King MRF, Brady KA, Galea S, Friedman SR, et al. Potential drivers of HIV acquisition in African-American women related to mass incarceration: an agent-based modelling study. BMC public health. 2018 dec;18(1):1387.

[3] Bershteyn A, Klein DJ, Eckhoff PA. Age-dependent partnering and the HIV transmission chain: a microsimulation analysis. Journal of the Royal Society, Interface. 2013 nov;10(88):20130613. Available from: http://­rsif.royalsocietypublishing.org/­cgi/­doi/­10.1098/­rsif.2013.0613.

[4] Borre ED, Hyle EP, Paltiel AD, Neilan AM, Sax PE, Freedberg KA, et al. The Clinical and Economic Impact of Attaining National HIV/AIDS Strategy Treatment Targets in the United States. Journal of Infectious Diseases. 2017 oct;216(7):798–807.

[5] Brookmeyer R, Boren D, Baral SD, Bekker LG, Phaswana-Mafuya N, Beyrer C, et al. Combination HIV prevention among MSM in South Africa: results from agent-based modeling. PloS one. 2014;9(11):e112668.

[6] Cambiano V, Miners A, Dunn D, McCormack S, Ong KJ, Gill ON, et al. Cost-effectiveness of pre-exposure prophylaxis for HIV prevention in men who have sex with men in the UK: a modelling study and health economic evaluation. The Lancet Infectious Diseases. 2018 jan;18(1):85–94. Available from: http://­linkinghub.elsevier.com/­retrieve/­pii/­S1473309917305406.

[7] Cameron E, Battle KE, Bhatt S, Weiss DJ, Bisanzio D, Mappin B, et al. Defining the relationship between infection prevalence and clinical incidence of Plasmodium falciparum malaria. Nature Communications. 2015 dec;6(1):8170. Available from: http://­www.nature.com/­articles/­ncomms9170.

[8] Chang ST, Chihota VN, Fielding KL, Grant AD, Houben RM, White RG, et al. Small contribution of gold mines to the ongoing tuberculosis epidemic in South Africa: a modeling-based study. BMC medicine. 2018 apr;16(1):52.

[9] Chen TM, Zhang SS, Feng J, Xia ZG, Luo CH, Zeng XC, et al. Mobile population dynamics and malaria vulnerability: a modelling study in the China-Myanmar border region of Yunnan Province, China. Infectious diseases of poverty. 2018 apr;7(1):36.

[10] Ciaranello AL, Morris BL, Walensky RP, Weinstein MC, Ayaya S, Doherty K, et al. Validation and calibration of a computer simulation model of pediatric HIV infection. PloS one. 2013 dec;8(12):e83389. Available from: http://­dx.plos.org/­10.1371/­journal.pone.0083389.

[11] de Vos AS, Prins M, Coutinho RA, van der Helm JJ, Kretzschmar MEEE. Treatment as prevention among injecting drug users; extrapolating from the Amsterdam cohort study. AIDS (London, England). 2014 mar;28(6):911–918. Available from: https://­insights.ovid.com/­crossref?an=00002030-201403270-00013.

[12] Eckhoff P. Mathematical models of within-host and transmission dynamics to determine effects of malaria interventions in a variety of transmission settings. American Journal of Tropical Medicine and Hygiene. 2013 may;88(5):817–827.

[13] Eckhoff PA, Wenger EA, Godfray HCJ, Burt A. Impact of mosquito gene drive on malaria elimination in a computational model with explicit spatial and temporal dynamics. Proceedings of the National Academy of Sciences. 2017 jan;114(2):E255–E264. Available from: http://­www.pnas.org/­lookup/­doi/­10.1073/­pnas.1611064114.

[14] Escudero DJ, Lurie MN, Mayer KH, Weinreb C, King M, Galea S, et al. Acute HIV infection transmission among people who inject drugs in a mature epidemic setting. AIDS (London, England). 2016 oct;30(16):2537–2544.

[15] Escudero DJ, Lurie MN, Mayer KH, King M, Galea S, Friedman SR, et al. The risk of HIV transmission at each step of the HIV care continuum among people who inject drugs: a modeling study. BMC Public Health. 2017 dec;17(1):614. Available from: http://­bmcpublichealth.biomedcentral.com/­articles/­10.1186/­s12889-017-4528-9.

[16] Espindola AL, Varughese M, Laskowski M, Shoukat A, Heffernan JM, Moghadas SM, et al. Strategies for halting the rise of multidrug resistant TB epidemics: assessing the effect of early case detection and isolation. International Health. 2017 mar;9(2):80–90.

[17] Fu R, Gutfraind A, Brandeau ML. Modeling a dynamic bi-layer contact network of injection drug users and the spread of blood-borne infections. Mathematical biosciences. 2016 mar;273:102–113. Available from: http://­linkinghub.elsevier.com/­retrieve/­pii/­S0025556416000043.

[18] Gantenberg JR, King M, Montgomery MC, Galarraga O, Prosperi M, Chan PA, et al. Improving the impact of HIV pre-exposure prophylaxis implementation in small urban centers among men who have sex with men: An agent-based modelling study. PloS one. 2018;13(7):e0199915.

[19] Gerardin J, Ouedraogo AL, McCarthy KA, Eckhoff PA, Wenger EA, Ouédraogo AL, et al. Characterization of the infectious reservoir of malaria with an agent-based model calibrated to age-stratified parasite densities and infectiousness. Malaria Journal. 2015 jun;14(1):231.

[20] Gerardin J, Eckhoff P, Wenger EA. Mass campaigns with antimalarial drugs: A modelling comparison of artemether-lumefantrine and DHA-piperaquine with and without primaquine as tools for malaria control and elimination. BMC Infectious Diseases. 2015 mar;15(1):144.

[21] Goedel WC, King MRF, Lurie MN, Nunn AS, Chan PA, Marshall BDL. Effect of Racial Inequities in Pre-exposure Prophylaxis Use on Racial Disparities in HIV Incidence Among Men Who Have Sex With Men: A Modeling Study. Journal of acquired immune deficiency syndromes (1999). 2018 nov;79(3):323–329.

[22] Gopalappa C, Farnham PG, Chen YH, Sansom SL. Progression and Transmission of HIV/AIDS (PATH 2.0). Medical decision making : an international journal of the Society for Medical Decision Making. 2017 feb;37(2):224–233.

[23] Gopalappa C, Sansom SL, Farnham PG, Chen YH. Combinations of interventions to achieve a national HIV incidence reduction goal: insights from an agent-based model. AIDS (London, England). 2017 nov;31(18):2533–2539. Available from: http://­insights.ovid.com/­crossref?an=00002030-201711280-00011.

[24] Gray RT, Prestage GP, Down I, Ghaus MH, Hoare A, Bradley J, et al. Increased HIV testing will modestly reduce HIV incidence among gay men in NSW and would be acceptable if HIV testing becomes convenient. PloS one. 2013;8(2):e55449.

[25] Herbeck JT, Mittler JE, Gottlieb GS, Goodreau SM, Murphy JT, Cori A, et al. Evolution of HIV virulence in response to widespread scale up of antiretroviral therapy: a modeling study. Virus evolution. 2016 jul;2(2):vew028.

[26] Hontelez JAC, Lurie MN, Barnighausen T, Bakker R, Baltussen R, Tanser F, et al. Elimination of HIV in South Africa through expanded access to antiretroviral therapy: a model comparison study. PLoS medicine. 2013 oct;10(10):e1001534. Available from: http://­dx.plos.org/­10.1371/­journal.pmed.1001534.

[27] Huynh GH, Klein DJ, Chin DP, Wagner BG, Eckhoff PA, Liu R, et al. Tuberculosis control strategies to reach the 2035 global targets in China: The role of changing demographics and reactivation disease. BMC Medicine. 2015 apr;13(1):88.

[28] Jewell BL, Cremin I, Pickles M, Celum C, Baeten JM, Delany-Moretlwe S, et al. Estimating the cost-effectiveness of pre-exposure prophylaxis to reduce HIV-1 and HSV-2 incidence in HIV-serodiscordant couples in South Africa. PloS one. 2015;10(1):e0115511.

[29] Johnson LF, Geffen N. A Comparison of Two Mathematical Modeling Frameworks for Evaluating Sexually Transmitted Infection Epidemiology. Sexually transmitted diseases. 2016 mar;43(3):139–146. Available from: http://­content.wkhealth.com/­linkback/­openurl?sid=WKPTLP:landingpage&an=00007435-201603000-00001.

[30] Kasaie P, Andrews JR, Kelton WD, Dowdy DW. Timing of tuberculosis transmission and the impact of household contact tracing: An agent-based simulation model. American Journal of Respiratory and Critical Care Medicine. 2014 apr;189(7):845–852. Available from: http://­www.atsjournals.org/­doi/­abs/­10.1164/­rccm.201310-1846OC.

[31] Kasaie P, Pennington J, Shah MS, Berry SA, German D, Flynn CP, et al. The Impact of Preexposure Prophylaxis Among Men Who Have Sex With Men: An Individual-Based Model. Journal of acquired immune deficiency syndromes (1999). 2017 jun;75(2):175–183. Available from: http://­insights.ovid.com/­crossref?an=00126334-201706010-00005.

[32] Kasaie P, Berry SA, Shah MS, Rosenberg ES, Hoover KW, Gift TL, et al. Impact of Providing Preexposure Prophylaxis for Human Immunodeficiency Virus at Clinics for Sexually Transmitted Infections in Baltimore City: An Agent-based Model. Sexually transmitted diseases. 2018 dec;45(12):791–797.

[33] Kessler J, Nucifora K, Li L, Uhler L, Braithwaite S. Impact and Cost-Effectiveness of Hypothetical Strategies to Enhance Retention in Care within HIV Treatment Programs in East Africa. Value in health : the journal of the International Society for Pharmacoeconomics and Outcomes Research. 2015 dec;18(8):946–955. Available from: http://­linkinghub.elsevier.com/­retrieve/­pii/­S1098301515050731.

[34] Khademi A, Saure D, Schaefer A, Nucifora K, Braithwaite RS, Roberts MS. HIV Treatment in Resource-Limited Environments: Treatment Coverage and Insights. Value in health : the journal of the International Society for Pharmacoeconomics and Outcomes Research. 2015 dec;18(8):1113–1119. Available from: http://­linkinghub.elsevier.com/­retrieve/­pii/­S1098301515050718.

[35] Klein DJ, Eckhoff PA, Bershteyn A. Targeting HIV services to male migrant workers in southern Africa would not reverse generalized HIV epidemics in their home communities: A mathematical modeling analysis. International Health. 2015 mar;7(2):107–113.

[36] Knight GM, Gomez GB, Dodd PJ, Dowdy D, Zwerling A, Wells WA, et al. The Impact and Cost-Effectiveness of a Four-Month Regimen for First-Line Treatment of Active Tuberculosis in South Africa. PloS one. 2015 dec;10(12):e0145796. Available from: http://­dx.plos.org/­10.1371/­journal.pone.0145796.

[37] Knittel AK, Snow RC, Riolo RL, Griffith DM, Morenoff J. Modeling the community-level effects of male incarceration on the sexual partnerships of men and women. Social science & medicine (1982). 2015 dec;147:270–279. Available from: http://­linkinghub.elsevier.com/­retrieve/­pii/­S0277953615302136.

[38] Luo W, Katz DA, Hamilton DT, McKenney J, Jenness SM, Goodreau SM, et al. Development of an Agent-Based Model to Investigate the Impact of HIV Self-Testing Programs on Men Who Have Sex With Men in Atlanta and Seattle. JMIR Public Health and Surveillance. 2018 jun;4(2):e58. Available from: http://­publichealth.jmir.org/­2018/­2/­e58/­.

[39] Luz PM, Morris BL, Grinsztejn B, Freedberg KA, Veloso VG, Walensky RP, et al. Cost-effectiveness of genotype testing for primary resistance in Brazil. Journal of acquired immune deficiency syndromes (1999). 2015 feb;68(2):152–161.

[40] Marshall BDL, Goedel WC, King MRF, Singleton A, Durham DP, Chan PA, et al. Potential effectiveness of long-acting injectable pre-exposure prophylaxis for HIV prevention in men who have sex with men: a modelling study. The lancet HIV. 2018 sep;5(9):e498–e505.

[41] McCarthy KA, Wenger EA, Huynh GH, Eckhoff PA. Calibration of an intrahost malaria model and parameter ensemble evaluation of a pre-erythrocytic vaccine. Malaria Journal. 2015 jan;14(1):6.

[42] McCormick AW, Abuelezam NN, Rhode ER, Hou T, Walensky RP, Pei PP, et al. Development, calibration and performance of an HIV transmission model incorporating natural history and behavioral patterns: application in South Africa. PloS one. 2014 may;9(5):e98272. Available from: http://­dx.plos.org/­10.1371/­journal.pone.0098272.

[43] McCormick AW, Abuelezam NN, Fussell T, Seage GRr, Lipsitch M. Displacement of sexual partnerships in trials of sexual behavior interventions: A model-based assessment of consequences. Epidemics. 2017 sep;20:94–101. Available from: http://­linkinghub.elsevier.com/­retrieve/­pii/­S1755436517300749.

[44] McCreesh N, Andrianakis I, Nsubuga RN, Strong M, Vernon I, Mckinley TJ, et al. Universal test, treat, and keep: improving ART retention is key in cost-effective HIV control in Uganda. BMC Infectious Diseases. 2017 dec;17(1):322. Available from: http://­bmcinfectdis.biomedcentral.com/­articles/­10.1186/­s12879-017-2420-y.

[45] McCreesh N, Andrianakis I, Nsubuga RN, Strong M, Vernon I, McKinley TJ, et al. Improving ART programme retention and viral suppression are key to maximising impact of treatment as prevention - a modelling study. BMC infectious diseases. 2017 aug;17(1):557. Available from: http://­bmcinfectdis.biomedcentral.com/­articles/­10.1186/­s12879-017-2664-6.

[46] McCreesh N, Andrianakis I, Nsubuga RN, Strong M, Vernon I, McKinley TJ, et al. Choice of time horizon critical in estimating costs and effects of changes to HIV programmes. PLOS ONE. 2018 may;13(5):e0196480. Available from: http://­dx.plos.org/­10.1371/­journal.pone.0196480.

[47] Monteiro JFG, Galea S, Flanigan T, de Lourdes Monteiro M, Friedman SR, Marshall BD. Evaluating HIV prevention strategies for populations in key affected groups: the example of Cabo Verde. International journal of public health. 2015;60(4):457–466.

[48] Nakagawa F, Miners A, Smith CJ, Simmons R, Lodwick RK, Cambiano V, et al. Projected Lifetime Healthcare Costs Associated with HIV Infection. PloS one. 2015;10(4):e0125018.

[49] Nakagawa F, van Sighem A, Thiebaut R, Smith C, Ratmann O, Cambiano V, et al. A Method to Estimate the Size and Characteristics of HIV-positive Populations Using an Individual-based Stochastic Simulation Model. Epidemiology (Cambridge, Mass). 2016 mar;27(2):247–256. Available from: http://­content.wkhealth.com/­linkback/­openurl?sid=WKPTLP:landingpage&an=00001648-900000000-99096.

[50] Nakagawa F. An epidemiological modelling study to estimate the composition of HIV-positive populations including migrants from endemic settings Fumiyo Nakagawa , on behalf of the Writing Group on HIV Epidemiologic Estimates in Countries With Migrant Populations. AIDS. 2017;(June 2016):417–425.

[51] Nalugoda F, Serwadda D, Eller MA, Robb ML, Gray R, Kigozi G, et al. A transmission-virulence evolutionary trade-off explains attenuation of HIV-1 in Uganda. eLife. 2016 nov;5:1–32.

[52] Nguyen TD, Olliaro P, Dondorp AM, Baird JK, Lam HM, Farrar J, et al. Optimum population-level use of artemisinin combination therapies: A modelling study. The Lancet Global Health. 2015 dec;3(12):e758–e766. Available from: http://­linkinghub.elsevier.com/­retrieve/­pii/­S2214109X1500162X.

[53] Luong Nguyen LB, Yazdanpanah Y, Maman D, Wanjala S, Vandenbulcke A, Price J, et al. Voluntary Community Human Immunodeficiency Virus Testing, Linkage, and Retention in Care Interventions in Kenya: Modeling the Clinical Impact and Cost-effectiveness. Clinical Infectious Diseases. 2018 aug;67(5):719–726.

[54] Olney JJ, Braitstein P, Eaton JW, Sang E, Nyambura M, Kimaiyo S, et al. Evaluating strategies to improve HIV care outcomes in Kenya: a modelling study. The lancet HIV. 2016 dec;3(12):e592–e600.

[55] Patel AR, Kessler J, Braithwaite RS, Nucifora KA, Thirumurthy H, Zhou Q, et al. Economic evaluation of mobile phone text message interventions to improve adherence to HIV therapy in Kenya. Medicine. 2017 feb;96(7):e6078.

[56] Penny MA, Galactionova K, Tarantino M, Tanner M, Smith TA. The public health impact of malaria vaccine RTS,S in malaria endemic Africa: Country-specific predictions using 18 month follow-up Phase III data and simulation models. BMC Medicine. 2015 jul;13(1):170.

[57] Penny MA, Pemberton-Ross P, Smith TA. The time-course of protection of the RTS,S vaccine against malaria infections and clinical disease. Malaria Journal. 2015 nov;14(1):437.

[58] Phillips AN, Cambiano V, Nakagawa F, Brown AE, Lampe F, Rodger A, et al. Increased HIV incidence in men who have sex with men despite high levels of ART-induced viral suppression: analysis of an extensively documented epidemic. PloS one. 2013;8(2):e55312.

[59] Phillips AN, Cambiano V, Miners A, Lampe FC, Rodger A, Nakagawa F, et al. Potential impact on HIV incidence of higher HIV testing rates and earlier antiretroviral therapy initiation in MSM. AIDS (London, England). 2015 sep;29(14):1855–1862. Available from: http://­insights.ovid.com/­crossref?an=00002030-201509100-00014.

[60] Phillips AN, Cambiano V, Nakagawa F, Bansi-Matharu L, Sow PS, Ehrenkranz P, et al. Cost Effectiveness of Potential ART Adherence Monitoring Interventions in Sub-Saharan Africa. PloS one. 2016;11(12):e0167654.

[61] Pizzitutti F, Pan W, Barbieri A, Miranda JJ, Feingold B, Guedes GR, et al. A validated agent-based model to study the spatial and temporal heterogeneities of malaria incidence in the rainforest environment. Malaria Journal. 2015 dec;14(1):514.

[62] Pizzitutti F, Pan W, Feingold B, Zaitchik B, Alvarez CA, Mena CF, et al. Out of the net: An agent-based model to study human movements influence on local-scale malaria transmission. PLoS ONE. 2018 mar;13(3):e0193493. Available from: http://­dx.plos.org/­10.1371/­journal.pone.0193493.

[63] Prats C, Montañola-Sales C, Gilabert-Navarro JF, Valls J, Casanovas-Garcia J, Vilaplana C, et al. Individual-Based Modeling of Tuberculosis in a User-Friendly Interface: Understanding the Epidemiological Role of Population Heterogeneity in a City. Frontiers in Microbiology. 2016 jan;6(JAN):1564. Available from: http://­journal.frontiersin.org/­Article/­10.3389/­fmicb.2015.01564/­abstract.

[64] Rigby SW, Johnson LF. The relationship between intimate partner violence and HIV: A model-based evaluation. Infectious Disease Modelling. 2017 feb;2(1):71–89.

[65] Romero-Severson EO, Alam SJ, Volz E, Koopman J. Acute-stage transmission of HIV: effect of volatile contact rates. Epidemiology (Cambridge, Mass). 2013 jul;24(4):516–521.

[66] Sauboin CJ, Van Bellinghen LA, Van De Velde N, Van Vlaenderen I. Potential public health impact of RTS,S malaria candidate vaccine in sub-Saharan Africa: a modelling study. Malaria Journal. 2015 dec;14(1):524. Available from: http://­www.malariajournal.com/­content/­14/­1/­524.

[67] van Schalkwyk C, Moodley J, Welte A, Johnson LF. Are associations between HIV and human papillomavirus transmission due to behavioural confounding or biological effects? Sexually Transmitted Infections. 2018 aug;2:1–7.

[68] Schneider K, Gray RT, Wilson DP. A cost-effectiveness analysis of HIV preexposure prophylaxis for men who have sex with men in Australia. Clinical infectious diseases : an official publication of the Infectious Diseases Society of America. 2014 apr;58(7):1027–1034.

[69] Selvaraj P, Wenger EA, Gerardin J. Seasonality and heterogeneity of malaria transmission determine success of interventions in high-endemic settings: a modeling study. BMC infectious diseases. 2018 aug;18(1):413.

[70] Sharma M, Smith JA, Farquhar C, Ying R, Cherutich P, Golden M, et al. Assisted partner notification services are cost-effective for decreasing HIV burden in western Kenya. AIDS (London, England). 2018 jan;32(2):233–241.

[71] Shcherbacheva A, Haario H, Killeen GF. Modeling host-seeking behavior of African malaria vector mosquitoes in the presence of long-lasting insecticidal nets. Mathematical biosciences. 2018 jan;295:36–47.

[72] Shrestha S, Hill AN, Marks SM, Dowdy DW. Comparing drivers and dynamics of tuberculosis in California, Florida, New York, and Texas. American Journal of Respiratory and Critical Care Medicine. 2017 oct;196(8):1050–1059. Available from: http://­www.atsjournals.org/­doi/­10.1164/­rccm.201702-0377OC.

[73] Shrestha S, Chihota V, White RG, Grant AD, Churchyard GJ, Dowdy DW. Impact of Targeted Tuberculosis Vaccination Among a Mining Population in South Africa: A Model-Based Study. American journal of epidemiology. 2017 dec;186(12):1362–1369.

[74] Smit M, Olney J, Ford NP, Vitoria M, Gregson S, Vassall A, et al. The growing burden of noncommunicable disease among persons living with HIV in Zimbabwe. AIDS (London, England). 2018 mar;32(6):773–782.

[75] Smith JA, Sharma M, Levin C, Baeten JM, van Rooyen H, Celum C, et al. Cost-effectiveness of community-based strategies to strengthen the continuum of HIV care in rural South Africa: a health economic modelling analysis. The lancet HIV. 2015 apr;2(4):e159–68. Available from: http://­dx.doi.org/­10.1016/­S2352-3018(15)00016-8.

[76] Steen R, Hontelez JAC, Veraart A, White RG, de Vlas SJ. Looking upstream to prevent HIV transmission: can interventions with sex workers alter the course of HIV epidemics in Africa as they did in Asia? AIDS (London, England). 2014 mar;28(6):891–899.

[77] Stevens ER, Li L, Nucifora KA, Zhou Q, McNairy ML, Gachuhi A, et al. Cost-effectiveness of a combination strategy to enhance the HIV care continuum in Swaziland: Link4Health. PloS one. 2018;13(9):e0204245.

[78] Stevens ER, Nucifora KA, Zhou Q, Braithwaite RS, Cleland CM, Ritchie AS, et al. Cost-Effectiveness of Peer- Versus Venue-Based Approaches for Detecting Undiagnosed HIV Among Heterosexuals in High-Risk New York City Neighborhoods. Journal of acquired immune deficiency syndromes (1999). 2018 feb;77(2):183–192.

[79] Suen SCC, Bendavid E, Goldhaber-Fiebert JD. Disease control implications of India’s changing multi-drug resistant tuberculosis epidemic. PLoS ONE. 2014 mar;9(3):e89822. Available from: http://­dx.plos.org/­10.1371/­journal.pone.0089822.

[80] Suen SC, Bendavid E, Goldhaber-Fiebert JD. Cost-effectiveness of improvements in diagnosis and treatment accessibility for tuberculosis control in India. The International Journal of Tuberculosis and Lung Disease. 2015 sep;19(9):1115–1124. Available from: http://­openurl.ingenta.com/­content/­xref?genre=article&issn=1027-3719&volume=19&issue=9&spage=1115.

[81] Tuite AR, Gallant V, Randell E, Bourgeois ACC, Greer AL. Stochastic agent-based modeling of tuberculosis in Canadian Indigenous communities. BMC Public Health. 2017 jan;17(1):1–12.

[82] Vandewalle BB, Llibre JM, Parienti JJ, Ustianowski A, Camacho R, Smith C, et al. EPICE-HIV: An Epidemiologic Cost-Effectiveness Model for HIV Treatment. PloS one. 2016 feb;11(2):e0149007. Available from: http://­dx.plos.org/­10.1371/­journal.pone.0149007.

[83] Walensky RP, Borre ED, Bekker LG, Resch SC, Hyle EP, Wood R, et al. The Anticipated Clinical and Economic Impact of 90-90-90 in South Africa. Annals of internal medicine. 2016;165(5):325–333. Available from: https://­www.ncbi.nlm.nih.gov/­pmc/­articles/­PMC5012932/­pdf/­nihms784208.pdf.

[84] White MT, Walker P, Karl S, Hetzel MW, Freeman T, Waltmann A, et al. Mathematical modelling of the impact of expanding levels of malaria control interventions on Plasmodium vivax. Nature communications. 2018 aug;9(1):3300.
