# Supplementary material for: Calibration of individual-based models to epidemiological data: A systematic review
Source: PLoS Comput Biol. 2020 May 11;16(5):e1007893. doi: 10.1371/journal.pcbi.1007893 (PMC7241852; doi:10.1371/journal.pcbi.1007893)
Supplement: S2 Table — (DOCX) [file pcbi.1007893.s002.docx]

**S2 Table. Description of calibration algorithms**

| Group | Algorithm | Brief description | Result | Reference |
| --- | --- | --- | --- | --- |
| Optimization | Grid search | In grid search, the researcher first divides each parameter range into a grid of parameter values. E.g. for a two-parameter space, the grid consists of all parameter combinations on the grid points for each of the two parameters. The simulation model is run for each parameter combination, and the “best fitting” parameter combination is chosen according to the goodness-of-fit (GOF) measure. | Single best parameter combination | [1] |
| Optimization | Latin hypercube | Urban et al. explain: “Latin hypercubes (LH) are constructed to avoid the collapsing property of grids: no two LH design points share the same value for any parameter. In the case of a two-dimensional design (Latin square), this property is equivalent to each row and each column containing exactly one design point. Unlike grids, Latin hypercubes ensemble sizes need not grow exponentially with the dimensionality of the parameter space, permitting a LH design to explore more parameters than a grid using the same ensemble size.” | Single best parameter combination | [2] |
| Optimization | Computer optimization methods (E.g. Nelder-Mead, coordinate descent with golden section search, Interior point algorithm) | Algorithmic optimization is a numerical method to find the minimum or maximum of a goodness-of-fit (GOF) measure for a multidimensional parameter space. Examples of calibration methods that routinely use algorithmic optimization include least-squares estimation, in which the squared distance is minimized; Maximum-likelihood estimation, in which the likelihood is maximized. Most algorithms of this type start at a random place in the parameter space and have an algorithm for moving towards the maximum (or minimum) until a convergence criterium is satisfied. | Single best parameter combination + optionally a confidence interval (using the hessian matrix). | [3] |
| Sampling algorithm | Rejection-Approximate Bayesian Computation (ABC) | ABC algorithms are simulation-based methods to approximate the posterior parameter distribution. ABC starts by sampling from prior distributions for the parameters under calibration. The rejection-ABC algorithm consists of running the simulation-model for each sampled parameter combination. Consequently, the model output is compared to the target statistics. The distance between the model-output and the target statistics is quantified using a GOF measure. The GOF measure for each parameter combination is then compared to a tolerance (ε), all parameter combinations with a GOF < ε are accepted. The accepted parameter combinations form the approximate posterior. Several extensions of rejection-ABC have been proposed to improve efficiency. | A sample from the approximate posterior distribution for parameter values | [4] [5]  [6] |
| Sampling algorithm | History matching | History matching with model emulation does not attempt to approximate the posterior (i.e. it does not make probabilistic statements about the parameter combinations) but is designed to reduce the parameter-search space. So strictly speaking, history matching is not a calibration method, but a pre-calibration method designed to reduce computational time for complex models. The user specifies an initial design, consisting of a grid of parameter combinations (e.g. maximin Latin hypercube design). Consequently, the simulation-model is run k times for each of these parameter combinations to produce model-outputs. Then, *emulator models* (see separate explanation below) are specified, e.g. regression models that take the model outputs as the dependent variables and the parameter combinations as explanatory variables. After this, the emulator model is used to interpolate an implausibility measure (a goodness-of-fit for emulated outcomes) for all parameter combinations within the space demarcated by the initial design space. A parameter combination with a *small value of the implausibility* measure indicates that a simulation run for the current parameter combination is likely to produce model-output that resembles the target statistics. Next, the algorithm checks whether the design parameter combinations are within the non-implausible space. If the design parameter combinations are not within the non-implausible space, the implausible regions of the parameter space are rejected, and a new wave of parameter combinations are drawn from within the non-implausible space. This process can have multiple waves until the algorithm reaches acceptance of a pre-defined number of parameter combinations. | Non-implausible region | [7] |
| Sampling algorithm | Bayesian melding | Alkema et al. explain: “In Bayesian melding, “melding” refers to combining information about the inputs and outputs of a model. Combining prior distributions with the likelihood (updating prior beliefs) gives the “posterior” distribution of the quantity of interest. Melding the prior distributions on inputs and output with the likelihood on output gives posterior distributions on inputs as well as outputs. The Bayesian melding approach produces a sample from the posterior distributions on inputs and outputs.” | A sample from the approximate posterior distributions on inputs and outputs | [8] |
|  | Model emulation | Emulation models are surrogates to replace the simulation model. To build the model emulator, the simulation-model is run to produce model-outputs for each parameter combination in the parameter range. The most basic form of an emulation model is a regression model that takes the model outputs as the dependent variables and the parameter combinations as explanatory variables. Model emulation can save computational time in case of complex simulation models that are computationally expensive to run. |  | [9][10] |
| Sampling algorithm | Markov chain Monte Carlo (MCMC) | MCMC methods are used to approximate the posterior distribution of the parameters of interest by random sampling from the parameter space. Many different versions of MCMC methods exist, we will explain the general principle behind these methods. MCMC methods start by picking a random parameter combination from the prior distribution. The model is run for this parameter value and the likelihood of the data given the model is calculated. Next, a new parameter is picked as a random value from a chosen proposal distribution around the current parameter combination (Monte Carlo part), again the likelihood for this value is calculated. Now both these likelihoods are compared and the algorithm jumps to (i.e. accepts into the posterior distribution) the new parameter combination with a probability determined by the size of the new parameter values likelihood compared to the current values likelihood (the Markov chain part). “As defined in probability theory, a Markov chain is a sequence of random variables θ1, θ2, . . ., for which, for any t, the distribution of θt given all previous θ’s depends only on the most recent value, θt−1.” When MCMC sampling converges, we have a sample from the posterior distribution. | A sample from the approximate posterior distribution for parameter values | [11] |
| Resampling step Bayesian calibration | Sampling Importance Resampling (SIR) | SIR is a method for sampling from the posterior. The first step (Sampling) is to draw a sample from the prior. The second step (Importance) involves calculating the measure of goodness-of-fit (e.g. the likelihood) for each of the parameter combinations drawn in step one. This measure of GOF is then used to compute an importance weight for each parameter combination. In the example of the likelihood, each parameter combination gets a weight proportional to the likelihood of the target statistics given the model output produced by running the model for this parameter combination. So the parameter combination with the biggest likelihood gets the largest weight. In the third and final step, we draw a sample with replacement from the parameter combinations obtained in step one, using the weights obtained in step two. The result is a sample from the posterior. SIR was found to be inefficient in many practical applications. Therefore more efficient methods such as *Incremental Mixture Importance Sampling* (*IMIS*, see below) have been developed. | A sample from the approximate posterior distribution | [12][13] |
| Resampling step Bayesian calibration | Incremental Mixture Importance Sampling (IMIS) | Similar to SIR, IMIS is a method for sampling from the posterior. The first step of IMIS consists of the SIR algorithm described above. In the second, iterative step, a multivariate normal distribution is constructed with the parameter combination with the maximum weight (i.e. maximum likelihood) from the first step as its centre. Then a new sample is drawn from the resulting multivariate normal distribution, and the process repeats itself until a convergence criterion is satisfied. After convergence, a resampling step similar to the resampling step used in SIR is used to obtain a sample from the posterior. | A sample from the approximate posterior distribution | [14] |

**References**

[1] Gilli M, Maringer D, Schumann E. Numerical methods and optimization in finance. Academic Press; 2019.

[2] Urban NM, Fricker TE. A comparison of Latin hypercube and grid ensemble designs for the multivariate emulation of an Earth system model. Computers & Geosciences. 2010;36(6):746–755.

[3] Nelder JA, Mead R. A simplex method for function minimization. The computer journal. 1965;7(4):308–313.

[4] Beaumont MA. Approximate Bayesian computation in evolution and ecology. Annual review of ecology, evolution, and systematics. 2010;41:379–406.

[5] Bertorelle G, Benazzo A, Mona S. ABC as a flexible framework to estimate demography over space and time: some cons, many pros. Molecular ecology. 2010;19(13):2609–2625.

[6] Csilléry K, Blum MG, Gaggiotti OE, François O. Approximate Bayesian computation (ABC) in practice. Trends in ecology & evolution. 2010;25(7):410–418.

[7] McKinley TJ, Vernon I, Andrianakis I, McCreesh N, Oakley JE, Nsubuga RN, et al. Approximate Bayesian Computation and simulation-based inference for complex stochastic epidemic models. Statistical science. 2018;33(1):4–18.

[8] Alkema L, Raftery AE, Brown T. Bayesian melding for estimating uncertainty in national HIV prevalence estimates. Sexually transmitted infections. 2008;84(Suppl 1):i11–i16.

[9] Oyebamiji OK, Wilkinson DJ, Li B, Jayathilake PG, Zuliani P, Curtis TP. Bayesian emulation and calibration of an individual-based model of microbial communities. Journal of computational science. 2019;30:194–208.

[10] Oâ€™Hagan A. Bayesian analysis of computer code outputs: A tutorial. Reliability Engineering & System Safety. 2006;91(10-11):1290–1300.

[11] Gelman A, Carlin JB, Stern HS, Dunson DB, Vehtari A, Rubin DB. Bayesian data analysis. Chapman and Hall/CRC; 2013.

[12] Menzies NA, Soeteman DI, Pandya A, Kim JJ. Bayesian methods for calibrating health policy models: a tutorial. Pharmacoeconomics. 2017;35(6):613–624.

[13] Rubin DB. Using the SIR algorithm to simulate posterior distributions. Bayesian statistics. 1988;3:395–402.

[14] Raftery AE, Bao L. Estimating and projecting trends in HIV/AIDS generalized epidemics using incremental mixture importance sampling. Biometrics. 2010;66(4):1162–1173.
