# Supplementary material for: Calibration of individual-based models to epidemiological data: A systematic review
Source: PLoS Comput Biol. 2020 May 11;16(5):e1007893. doi: 10.1371/journal.pcbi.1007893 (PMC7241852; doi:10.1371/journal.pcbi.1007893)
Supplement: S1 Text — (DOCX) [file pcbi.1007893.s003.docx]

**S1 Text. Obtaining parameter uncertainty using an optimisation algorithm, quoted from Sauboin et al. *[1]***

“**Capturing uncertainty and heterogeneity**

A main source of uncertainty comes from the observation of incidence rates and vaccine efficacy estimates of the Phase III trial. Although the Phase III trial is powered for clinical malaria incidence and severe malaria incidence overall, larger variations appear when stratifying data by malaria transmission level and periods of follow- up. In order to capture this uncertainty, model parameter distributions are generated by repeating the calibration process with several incidence rates. These incidence rates are randomly drawn from a log-normal distribution generated using median values and 95 % CI for each 6-month follow-up period and for each age-group. This produced a set of values for the calibrated parameters from which non-parametric distributions were derived. A random value is drawn from the corresponding corresponding non-parametric distribution for each parameter to simulate the model process for one individual. Table 2 shows the point estimates and CIs for the parameters. Additional file 1: Figure S1 shows the distributions for q parameters and Additional file 2: Figure S2 shows the distributions for parameters a1 to a6 and f1 to f6.”

**References**

[1] Sauboin CJ, Van Bellinghen LA, Van De Velde N, Van Vlaenderen I. Potential public health impact of RTS,S malaria candidate vaccine in sub-Saharan Africa: a modelling study. Malaria Journal. 2015 dec;14(1):524. Available from: http://­www.malariajournal.com/­content/­14/­1/­524.
