# Supplementary material for: Calibration of individual-based models to epidemiological data: A systematic review
Source: PLoS Comput Biol. 2020 May 11;16(5):e1007893. doi: 10.1371/journal.pcbi.1007893 (PMC7241852; doi:10.1371/journal.pcbi.1007893)
Supplement: S2 Text — (DOCX) [file pcbi.1007893.s004.docx]

**S2 Text. Selected quotes of rationales for choosing model calibration method**

**Cambiano *et al.* [1]**

“ The model was run >100,000 times, independently sampling the 20 parameter values relating to sexual behaviour, testing and transmission(1) from the distribution indicated in Supplementary Table 3. We recorded parameter sets which provided a fit value < 1.8, which represents a situation where on average the model outputs are within 12% of the data (sum of the weights = 1.49). To generate uncertainty bounds we present the median and 90% range (5%-95% centiles) over runs in this fit value range. 101 such parameter sets were identified. These parameter sets were used (by selecting from the 101 at random each time the model was run, always from 1980) when doing repeated runs to project forward to 2096. *Over 50,000 runs were performed, but only those in which the fit value was again < 1.8 were considered for the model projections (3,500).* For the main results we divided the distributions of the parameters sampled as part of the PSA into tertiles and calculated the mean and 90% range across the simulations with the same combination of PSA parameters tertiles. The only exception is Supplementary Figure 5 where we presented the value of each single simulation to illustrate run-to-run variability.”

**Goedel *et al*. [2]**

“An initial set of 100 iterations were run for 120 time-steps to compare the initial model output to the calibration targets. To further improve the ability of the model to reproduce such targets, an initial 500 iterations are run for 120 time-steps with a sweep over a set of scaling parameters that are applied to each of the following parameters: the frequency of sexual behavior (Factor: 2.0), the monthly probability of testing for HIV infection (Factor: 0.3) and the monthly probability of initiation of antiretroviral treatment (Factor: 0.4). *Model refinement was then conducted by adjusting key parameters for which there were greater levels of uncertainty in their values to minimize differences between the model output and available empirical data with regard to secondary calibration targets, where we used an iterative stepwise sweep of calibration parameters to approximate estimates of observed year-end prevalence of HIV infection.* Although this process does not guarantee the validity of the model, it does allow for the exclusion of parameter values that do not reproduce the available empirical data adequately. Specifically, these calibration targets were achieved by scaling parameters related to the number of sexual acts engaged in per-time step and condom use.”

**Huynh *et al*. [3]**

“All other parameters were informed by available literature and expert opinion, and were held fixed during the calibration. This reduces the total number of dimensions in the calibration parameter space to a computationally tractable size.”

**Kessler *et al*. [4]**

“Ultimately, *we chose to use visual inspection* because the survival curves did not fit closely enough using the other two more quantitative approaches.”

**Klein *et al*.** [5]

“coordinate descent with golden section search was used to incrementally adjust parameter values, one at a time, so as to minimize a squared error loss function. *This approach to calibration worked to a limited extent, but was unable to resolve co-varying parameters. These parameters were adjusted by hand in order to minimize the same loss function.* Subsequent versions of the EMOD-HIV model will employ a sophisticated calibration process that uses Bayesian methods.”

**McCreesh *et al*. [6]**

“The model was fitted to the empirical data using history matching with model emulation, which allowed uncertainties in model inputs and outputs to be fully represented, and allowed realistic estimates of uncertainty in model results to be obtained.”

**Nakagawa *et al*. [7]**

“ABC methods - allow us - to explore a wide parameter space and to consider multiple sets of parameter values which are consistent with the observed data, instead of converging to a single set of parameter values.”

**Nakagawa *et al*. [8]**

“the model is calibrated using aBc methods. The model naturally lends itself to working in a Bayesian framework to account for multiple parameter combinations providing outputs which fit well to the observed data (instead of converging to a single set of parameter values as would be the case in maximum likelihood estimation). aBc methods are suitable for calibrating simulation models to multiple datasets within tolerance bounds and have the advantage of accounting for parameter uncertainty and correlations. they are ideal for our purpose because we wish to explore a wide parameter space and consider as many param- eter sets as possible that are consistent with the data.”

**Olney *et al*. [9]**

“Due to the complex nature of the model and the range of data available from AMPATH for calibration, the cascade model was calibrated by hand.”

**Selvaraj *et al*. [10]**

“The likelihoods of each parameter set were calculated as described in [5, 29] using a Dirichlet-multinomial distribution to compare simulation data with field data.” … “Monthly EIR values were *adaptively tuned* for a given parameter set until there was a good fit of simulation data to the reference dataset (Fig. 1).” (page 3 of Selvaray, under calibration methodology)

**References**

[1] Cambiano V, Miners A, Dunn D, McCormack S, Ong KJ, Gill ON, et al. Cost-effectiveness of pre-exposure prophylaxis for HIV prevention in men who have sex with men in the UK: a modelling study and health economic evaluation. The Lancet Infectious Diseases. 2018 jan;18(1):85–94. Available from: http://­linkinghub.elsevier.com/­retrieve/­pii/­S1473309917305406.

[2] Goedel WC, King MRF, Lurie MN, Nunn AS, Chan PA, Marshall BDL. Effect of Racial Inequities in Pre-exposure Prophylaxis Use on Racial Disparities in HIV Incidence Among Men Who Have Sex With Men: A Modeling Study. Journal of acquired immune deficiency syndromes (1999). 2018 nov;79(3):323–329.

[3] Huynh GH, Klein DJ, Chin DP, Wagner BG, Eckhoff PA, Liu R, et al. Tuberculosis control strategies to reach the 2035 global targets in China: The role of changing demographics and reactivation disease. BMC Medicine. 2015 apr;13(1):88.

[4] Kessler J, Nucifora K, Li L, Uhler L, Braithwaite S. Impact and Cost-Effectiveness of Hypothetical Strategies to Enhance Retention in Care within HIV Treatment Programs in East Africa. Value in health : the journal of the International Society for Pharmacoeconomics and Outcomes Research. 2015 dec;18(8):946–955. Available from: http://­linkinghub.elsevier.com/­retrieve/­pii/­S1098301515050731.

[5] Klein DJ, Eckhoff PA, Bershteyn A. Targeting HIV services to male migrant workers in southern Africa would not reverse generalized HIV epidemics in their home communities: A mathematical modeling analysis. International Health. 2015 mar;7(2):107–113.

[6] McCreesh N, Andrianakis I, Nsubuga RN, Strong M, Vernon I, Mckinley TJ, et al. Universal test, treat, and keep: improving ART retention is key in cost-effective HIV control in Uganda. BMC Infectious Diseases. 2017 dec;17(1):322. Available from: http://­bmcinfectdis.biomedcentral.com/­articles/­10.1186/­s12879-017-2420-y.

[7] Nakagawa F, Miners A, Smith CJ, Simmons R, Lodwick RK, Cambiano V, et al. Projected Lifetime Healthcare Costs Associated with HIV Infection. PloS one. 2015;10(4):e0125018.

[8] Nakagawa F, van Sighem A, Thiebaut R, Smith C, Ratmann O, Cambiano V, et al. A Method to Estimate the Size and Characteristics of HIV-positive Populations Using an Individual-based Stochastic Simulation Model. Epidemiology (Cambridge, Mass). 2016 mar;27(2):247–256. Available from: http://­content.wkhealth.com/­linkback/­openurl?sid=WKPTLP:landingpage&an=00001648-900000000-99096.

[9] Olney JJ, Braitstein P, Eaton JW, Sang E, Nyambura M, Kimaiyo S, et al. Evaluating strategies to improve HIV care outcomes in Kenya: a modelling study. The lancet HIV. 2016 dec;3(12):e592–e600.

[10] Selvaraj P, Wenger EA, Gerardin J. Seasonality and heterogeneity of malaria transmission determine success of interventions in high-endemic settings: a modeling study. BMC infectious diseases. 2018 aug;18(1):413.
