# Supplementary material for: Calibration of individual-based models to epidemiological data: A systematic review
Source: PLoS Comput Biol. 2020 May 11;16(5):e1007893. doi: 10.1371/journal.pcbi.1007893 (PMC7241852; doi:10.1371/journal.pcbi.1007893)
Supplement: S1 Appendix — (DOCX) [file pcbi.1007893.s005.docx]

**S1 Appendix. Parameter search strategies by disease and year of publication.**

Table A below shows the parameter search strategy by year of publication. The p-value for the Fisher’s exact test was 0.4654, indicating that we cannot reject the null-hypothesis that the parameter search strategy is independent of the year of publication. Similar to a Chi-square test, Fisher's exact tests for independence of two categorical variables. Fisher’s exact test is more accurate than a chi-square test of independence when the expected numbers are small, as is the case in the table.

**Table A. Parameter search strategy by year of publication**

|  | 2013 | 2014 | 2015 | 2016 | 2017 | 2018 |
| --- | --- | --- | --- | --- | --- | --- |
| Informal/Unidentifiable | 2 | 4 | 11 | 10 | 8 | 9 |
| Optimization | 2 | 2 | 5 | 0 | 1 | 4 |
| Sampling | 3 | 1 | 6 | 3 | 6 | 7 |

Table B below shows the parameter search strategy (divided into reporting: yes/no) by year of publication. Where not reported consists of only the articles with unidentifiable parameter search strategies. The p-value for the Fisher’s exact test was 0.6506, indicating that we cannot reject the null-hypothesis that the reporting on calibration is independent of the year of publication.

**Table B. Reporting on a parameter search strategy (y/n) by year of publication**

|  | 2013 | 2014 | 2015 | 2016 | 2017 | 2018 |
| --- | --- | --- | --- | --- | --- | --- |
| Not reported (unidentifiable onlyl) | 2 | 4 | 6 | 6 | 7 | 7 |
| Reported (informal, optimization & sampling) | 5 | 3 | 16 | 7 | 8 | 13 |

Table C below shows the parameter search strategy by disease studied (HIV, TB, malaria). The p-value for the Fisher’s exact test was 0.1837, indicating that we cannot reject the null-hypothesis that the reporting on calibration is independent of the disease studied.

**Table C. Parameter search strategy by disease studied (i.e. HIV, TB, malaria)**

|  | HIV | TB | Malaria |
| --- | --- | --- | --- |
| Informal/Unidentifiable | 33 | 3 | 8 |
| Optimization | 9 | 4 | 1 |
| Sampling | 16 | 3 | 7 |
