# Supplementary material for: Calibration of individual-based models to epidemiological data: A systematic review
Source: PLoS Comput Biol. 2020 May 11;16(5):e1007893. doi: 10.1371/journal.pcbi.1007893 (PMC7241852; doi:10.1371/journal.pcbi.1007893)
Supplement: S2 Appendix — (DOCX) [file pcbi.1007893.s006.docx]

**S2 Appendix. Histograms and plots for counts of target statistics, calibrated parameters and the size of the simulated population**

| **Figure A. Histogram of the number of target statistics.**  **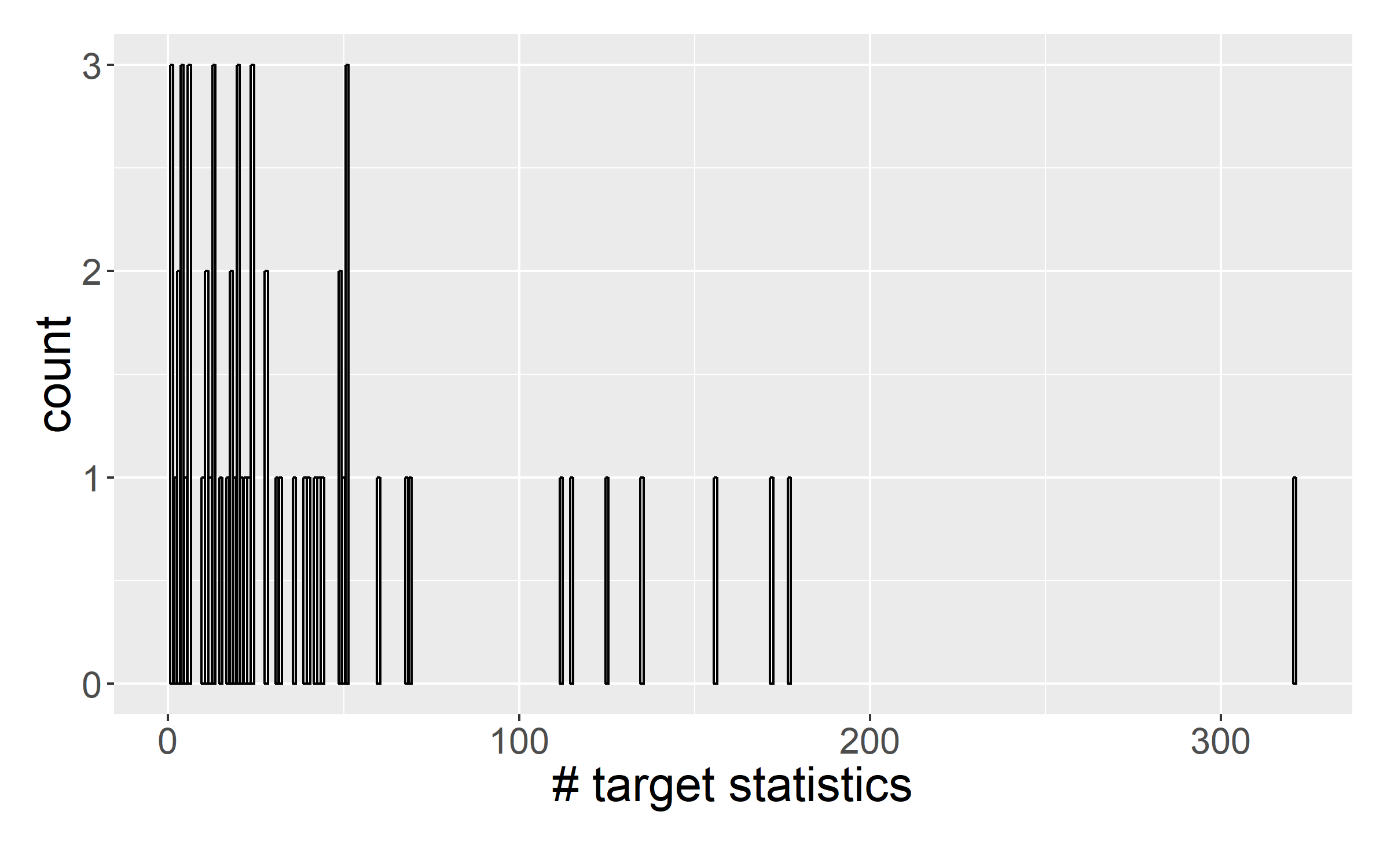** |
| --- |

**Figure B. Histogram of the number of calibrated parameters**

**
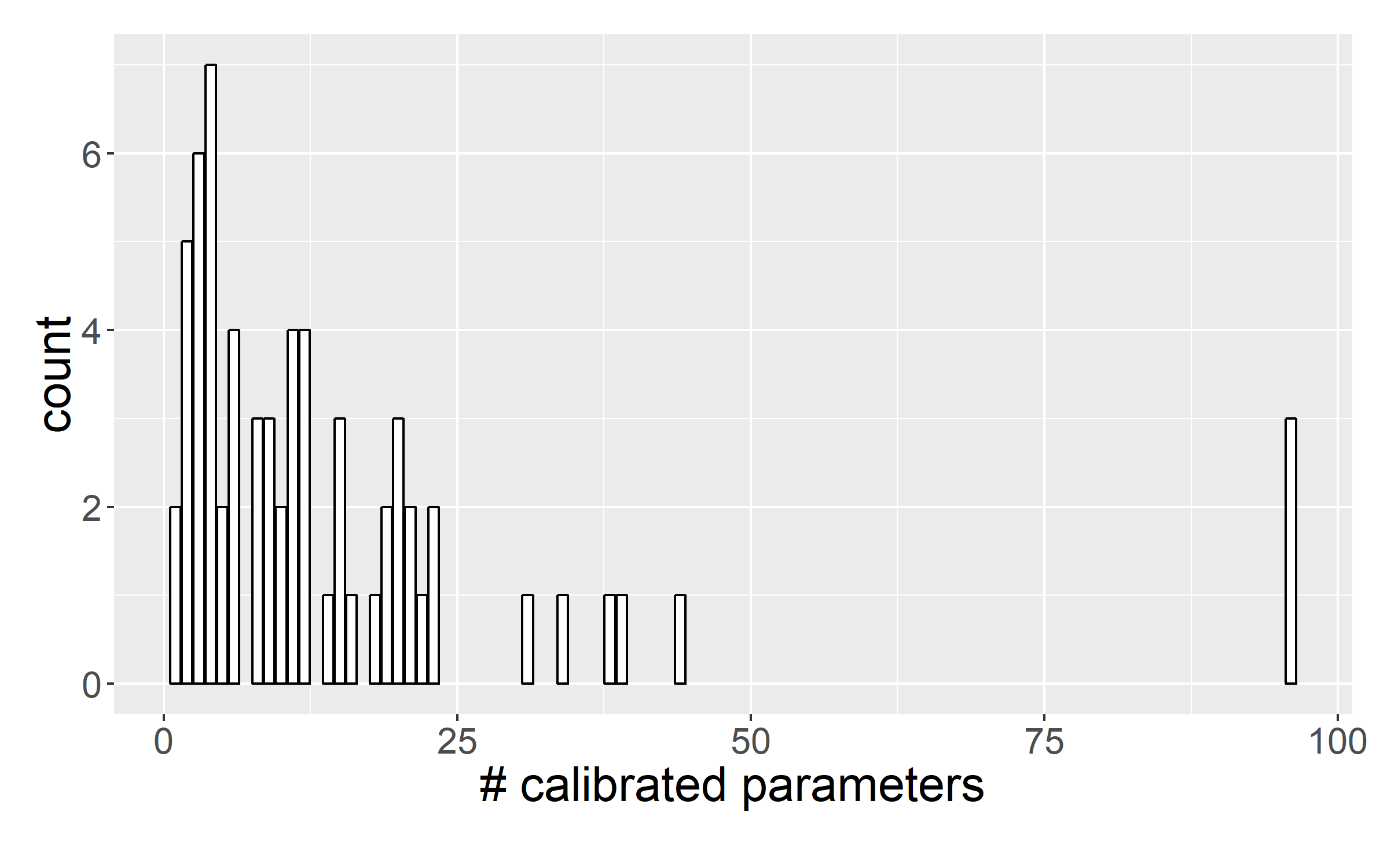
**

**Figure C. Plot of the number of target statistics against the number of calibrated parameters by parameter search strategy (colour).** The yellow line indicates the diagonal, points above the yellow line indicate articles in which the number of calibrated parameters exceeded the number of target statistics.

**
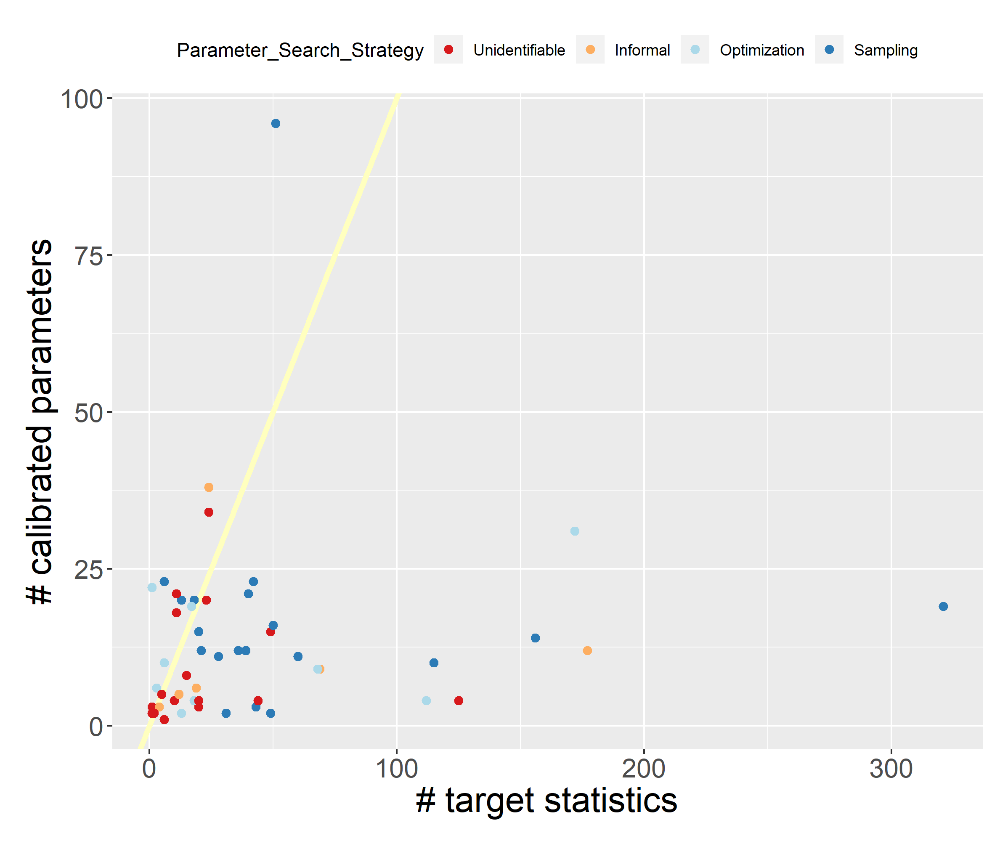
**

**Figure D. Histogram of log10 of the size of the simulated population.**

**
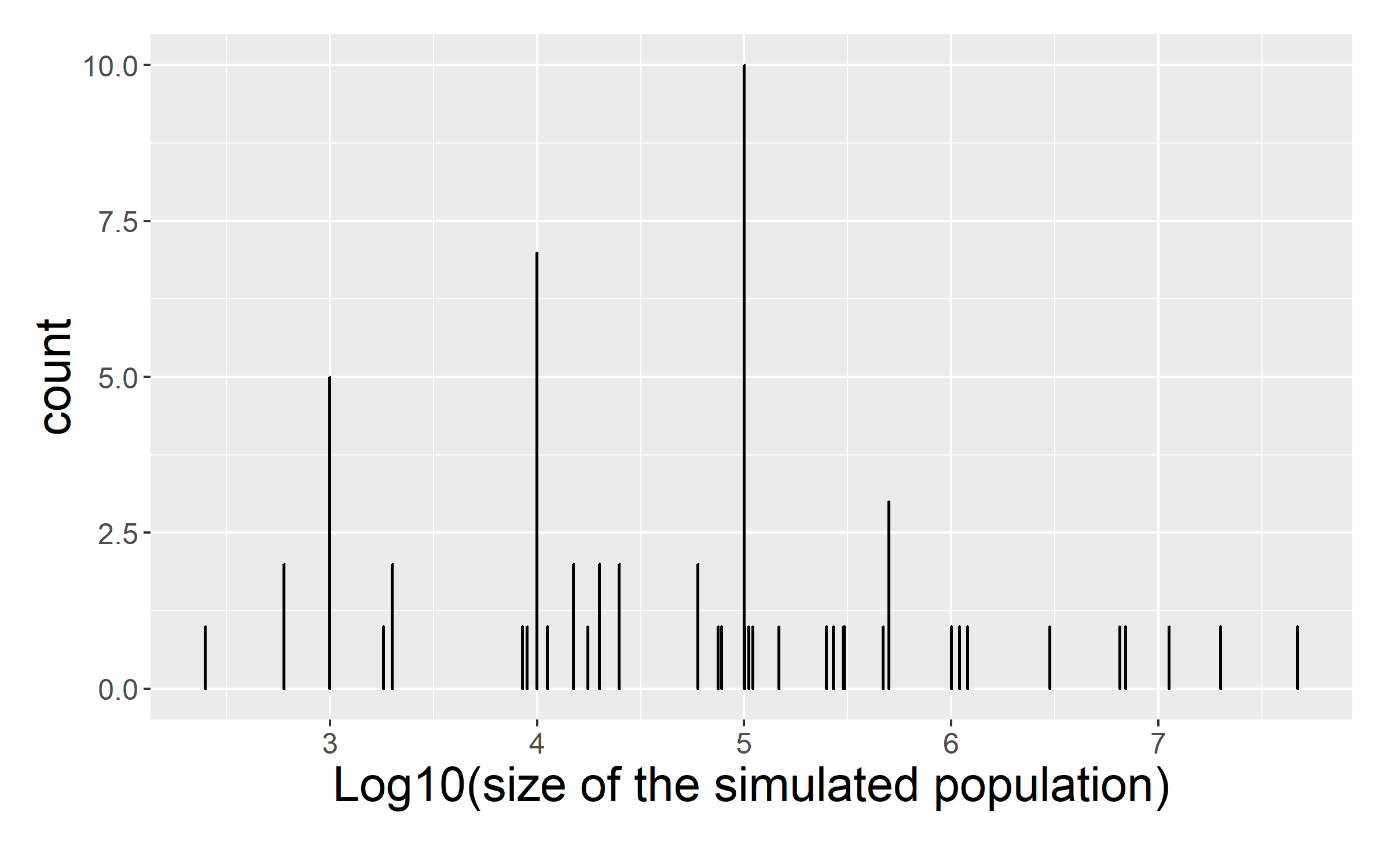
**
