# Supplementary material for: Calibration of individual-based models to epidemiological data: A systematic review
Source: PLoS Comput Biol. 2020 May 11;16(5):e1007893. doi: 10.1371/journal.pcbi.1007893 (PMC7241852; doi:10.1371/journal.pcbi.1007893)
Supplement: S3 Appendix — (DOCX) [file pcbi.1007893.s007.docx]

**Calibration reporting checklist**

| **Section/topic** | **#** | **Checklist item** | **Reported on page** |
| --- | --- | --- | --- |
| **CONTEXTUAL VARIABLES** | | | |
| **Target data (for each different source of target data, e.g. mortality, incidence, etc.)** | | | |
| Description | 1 | Describe the type of the target data (e.g. annual age-group-specific mortality rate). |  |
| Type of study for the data source | 2 | Describe the design of the study from which the data was obtained (e.g. observational study, randomised controlled trial). |  |
| Description of source | 3 | Describe how the individual data points were obtained (e.g. incidence is based on a statistical estimation with a certain sample size). |  |
| The number of data points | 4 | Provide the number of data points for this target data source. |  |
| Time-series | 5 | Describe whether the target data are a time-series (e.g. mortality data for the years 2000 until 2016). |  |
| **Parameters (for each parameter clearly indicate below topics, for example in a table)** | | | |
| **Information available before calibration** | | | |
| Calibrated | 6 | Indicate whether the current parameter is calibrated. |  |
| Prior distribution range | 7 | Describe the prior distribution range for this parameter. Preferably the range with accompanying distribution and point estimate, if applicable. |  |
| The source for the prior distribution | 8 | Provide the reference for the study from which the prior was obtained. |  |
| **Information available after calibration** | | | |
| Obtained posterior parameter distribution | 9 | Describe the obtained posterior parameter distribution (e.g. single best parameter combination, single best parameter combination with a confidence interval, or posterior distribution). |  |
| Posterior point estimate | 10 | Provide the point estimate for the parameter. |  |
| Posterior distribution range | 11 | If applicable, provide the posterior distribution range for this parameter. Preferably in the table next to the prior range. |  |
| Plots of the posterior distribution *(optional)* | 12 | Plots of posteriors for each parameter against all other parameters could be provided in the appendix. |  |
| **Size of the population** | | | |
| Size of the population of inference | 13 | Provide the size of the population of inference (e.g. the entire Dutch population, 17.18 million). |  |
| Size of the simulated population | 14 | Provide the number of individuals simulated in the individual-based model. |  |
| Method of scale-up *(Optional if the size of the population of inference differs from the size of the simulated population)* | 15 | Describe how the effect in the simulated population was scaled up to the size of the population of inference (E.g. the model was run a number of times and outcomes were averaged over multiple simulation runs per parameter set). |  |

| **Section/topic** | **#** | | **Checklist item** | | | **Reported on page** |
| --- | --- | --- | --- | --- | --- | --- |
| **CALIBRATION METHOD** | | | | | | |
| **Parameter search strategy (choose between Optimization or Sampling methods)** | | | | | | |
| **Optimisation method** | | 16 | | Describe the specific optimisation method used (e.g. Grid search, iterative descent-guided optimisation). |  | |
| Optimisation algorithm *(only applicable for iterative descent-guided optimisation)* | | 17 | | Describe the specific algorithm used for optimisation (e.g. Nelder-Mead, Coordinate-descent with golden section search). |  | |
| Reason for a specific method | | 18 | | Describe the choice process that leads to picking this particular method (e.g. Performs well given the number of target statistics and parameters according to a particular methods comparison paper). |  | |
| Initial values for parameters | | 19 | | Provide the initial values for parameters (i.e. starting point for the optimisation procedure). In case starting values were drawn at random by the algorithm, provide the random seed entered before running the algorithm. |  | |
| Check for local maximum | | 20 | | Provide information on whether different sets of initial values led to the same calibration result. |  | |
| Random number generator & seed | | 21 | | Provide the random number generator/ software used and the seed provided. |  | |
| Stopping rule | | 22 | | Report the absolute or relative convergence tolerance; this can also be the default for the algorithm you are using in which case “default” will suffice. |  | |
| **Sampling method** | | 23 | | Describe the sampling method used (e.g. Rejection ABC, Bayesian calibration). |  | |
| Specifics sampling method | | 24 | | Provide the specifics for how parameter combinations are sampled (e.g. Markov chain Monte Carlo, Latin hypercube, random draw from prior, Sampling Importance Resampling (SIR), Incremental Mixture Importance Sampling (IMIS)). |  | |
| Reason for choice of a specific method | | 25 | | Describe the choice process that led to picking this particular method (e.g. Performs well give the number of target statistics and parameters according to a particular methods comparison paper). |  | |
| Random number generator | | 26 | | Provide the random number generator/ software used and the seed provided. |  | |
| Number of model runs | | 27 | | Report the number of times the algorithm ran the model. |  | |
| Acceptance criteria *(Only applicable if the method involves acceptance criteria)* | | 28 | | Report the threshold for the GOF under which parameter combinations are accepted/retained. |  | |
| Goodness-of-fit (GOF) measure | | | | | | |
| GOF measure | | 29 | | Provide the GOF measure used (e.g. absolute distance, relative distance, surrogate likelihood). |  | |
| Weighted GOF measure *(Optional)* | | 30 | | Provide the computation of the total GOF (i.e. the sum of the separate data points over all different sources of target data) if the separate parts were weighted differently (e.g. mortality weighted 20% of the total GOF while incidence weighted 80% of the total score). |  | |
