# Supplementary material for: Calibration of individual-based models to epidemiological data: A systematic review
Source: PLoS Comput Biol. 2020 May 11;16(5):e1007893. doi: 10.1371/journal.pcbi.1007893 (PMC7241852; doi:10.1371/journal.pcbi.1007893)
Supplement: Attachment — Submitted filename: reviewer.docx [file pcbi.1007893.s008.docx]

Dear authors,

Thanks for your great work on ‘Fitting individual-based models to data in HIV, tuberculosis and malaria: a systematic review’. The systematic review looks into the calibration of individual-based models (IBMs). This review indicates that there is still a gap in the reporting of calibration and validation techniques. Also, it encourages others to compare different calibration techniques available for IBMs

Please find my comments below:

Abstract

- A lot of repetitive sentences in abstract
- Consistency of numbering not uniform (eg: Thirty-two instead of 32)

Figure 1

- Show studies included in between the phases

Figure 2

- Can be more consistent with the text

Table 2

- Not formatted. Some text is hidden (Suen et al.)
- Are the table rows arranged randomly?

Methods

- In the methods section, the authors mention the use of calibration Reporting Checklist of Stout et al. and say ‘it was extended by several items’. But they do not specify what they did.

Results

- Squared distance is used in 6/14 (Check the table again). Inconsistency between table and text.
- Authors do not specify the commonly used GOF for sampling algorithm unlike optimization algorithm. Is that intentional?
- Target data and number of calibrated parameters: there is a huge range to be of any meaningful inference. Authors could have given one of the central tendencies (if possible) instead of range. Also, authors mention ‘The number of target statistics varied from one to around 150.’ Why is it around 150? Is it not clear from the article?
- Authors mention that ‘For several articles, the number of calibrated parameters appeared to exceed the number of target statistics.’ But do not specify how many.
- Numbering format could be improved throughout the text.
- Authors mention ‘The number of articles selected by publication year increased from seven in 2013 to 20 in 2018’. It might be interesting to look at the number of articles among these which reported calibration.
- Except this sentence ‘Only 32 (38%) articles mentioned that a validation of the model had been performed’ being used several times, one would not see any information about validation.

Discussion

- Can be read ambiguously ‘While algorithmic parameter-search strategies favour reproducibility, unclear or incomplete reporting, and non-disclosure of software code, can still prevent reproducibility’
- Repetitive ‘These issues suggest that there is a need for research comparing the performance of calibration methods to inform decisions about the choice of parameter-search strategy and GOF’ and ‘Our review serves as a primer for methodological studies aimed at understanding the relative performance of alternative calibration methods.’
- ‘Authors say ‘Manual adjustment of parameter values and visual inspection of GOF, on the other hand, does not favour reproducibility ... Nevertheless, such informal methods may perform equally ... and can be useful for simple didactic purposes.’ Would that mean manual adjustments do not favour reproducibility and yet one can use it? What should the readers infer from this?
