# Supplementary material for: Calibration of individual-based models to epidemiological data: A systematic review
Source: PLoS Comput Biol. 2020 May 11;16(5):e1007893. doi: 10.1371/journal.pcbi.1007893 (PMC7241852; doi:10.1371/journal.pcbi.1007893)
Supplement: Attachment — Submitted filename: Rebuttal.docx [file pcbi.1007893.s009.docx]

**Reviewer #1:**

Abstract

- A lot of repetitive sentences in abstract

***Response:*** *We removed the repetitive sentences from the abstract. The last part of the abstract, in which most of the repetition took place, now reads: “Reporting on calibration methods is far from optimal in epidemiological modelling studies of HIV, malaria and TB transmission dynamics. There is a need for research comparing the performance of calibration methods to inform decisions about the choice of parameter-search strategy and GOF measure. The adoption of better documented, algorithmic calibration methods could improve both reproducibility and the quality of inference in model-based epidemiology.”*

- Consistency of numbering not uniform (eg: Thirty-two instead of 32)

***Response:*** *We thank the reviewer for pointing out inconsistencies in numbering, we now follow the conventions at* [*https://www.aje.com/arc/editing-tip-using-numbers-scientific-manuscripts/*](https://www.aje.com/arc/editing-tip-using-numbers-scientific-manuscripts/) *throughout the manuscript. For example, we changed the example provided by the reviewer as follows: “Model validation was reported in 32 (38%) articles.”*

Figure 1

- Show studies included in between the phases

***Response:*** *We thank the reviewer for pointing out the missing box in figure 1, we added the corresponding box to the figure.*

Figure 2

- Can be more consistent with the text

***Response:*** *We edited figure 2 to be more consistent with the text.*

Table 2

- Not formatted. Some text is hidden (Suen et al.)
- Are the table rows arranged randomly?

***Response:*** *We formatted table 2 according to make all text visible. We incorporated the sorting (according to sampling algorithm) in the header of both tables 1 & 2.*

Methods

- In the methods section, the authors mention the use of calibration Reporting Checklist of Stout et al. and say ‘it was extended by several items’. But they do not specify what they did.

***Response:*** *We added a sentence to explain this:* ***“****This form was based on the Calibration Reporting Checklist of Stout et al. and was extended by several items, including; the software and hardware used to build the model, the size of the initial population of agents and the name of the modelling platform. Additionally, we inserted several items to collect information on the number of calibrated parameters, the number of fixed parameters, and the number of targets. We noted how information on these counts was reported in the articles (i.e. the number was explicitly provided, could be deduced from text or figures, was provided incompletely or was not provided).”*

Results

- Squared distance is used in 6/14 (Check the table again). Inconsistency between table and text.

***Response:*** *We thank the reviewer for pointing out this inconsistency, we corrected the value to 6/14.*

- Authors do not specify the commonly used GOF for sampling algorithm unlike optimization algorithm. Is that intentional?

***Response:*** *This inconsistency was unintentional; we provide the resulting sentence below:*

*“Most articles (10/26) used the approximate likelihood as a measure of GOF, and various GOF measures were used in the remaining articles, these include absolute distances (4/26), relative distances (4/26) and squared distances (4/26). (see Table 2).”*

- Target data and number of calibrated parameters: there is a huge range to be of any meaningful inference. Authors could have given one of the central tendencies (if possible) instead of range. Also, authors mention ‘The number of target statistics varied from one to around 150.’ Why is it around 150? Is it not clear from the article?”

***Response:*** *The reviewer is right; it was often unclear from the articles how many target statistics and calibrated parameters were involved. For that reason, we set out to collect extra information: “We inserted several items to collect information on the number of calibrated parameters, the number of fixed parameters, and the number of targets. We noted how information on these counts was reported in the articles (i.e. the number was explicitly provided, could be deduced from text or figures, was provided incompletely or was not provided).” To illustrate that it is often difficult and sometimes unclear from the articles how much a specific count was we added the following sentence to the limitations section in the discussion: “Another limitation is that the counts presented in this review often had to be deduced from the article, this was a difficult and laborious task involving manual counting of target statistics in either the text, figures or tables, a process that is prone to error.” We then continued to provide more information on these counts in the results:*

*“The number of target statistics, the number of calibrated parameters and the size of the simulated population*

*The number of target statistics was explicitly mentioned in only three (4%) of the 84 included articles, for 62 (74%) articles we had enough information to attempt to deduce this number from either text or figures. The remaining 19 (23%) articles either provided incomplete information (11/19) or no information (8/19). Some (4/65) of the articles for which we were able to obtain the number of target statistics had different numbers of target statistics for calibration in different locations or calibration to different diseases. The 61 (73%) articles for which we were able to obtain a single count had a median number of target statistics of 23 (range 1 – 321). A histogram of the number of target statistics is provided in figure A in S2 Appendix.*

*The number of calibrated parameters was explicitly mentioned in 11 (13%) of the 84 included articles, for another 53 (63%) articles it was possible to deduce this number from either text or figures. The remaining 20 (24%) articles either provided incomplete information (10/20) or no information at all (10/20). The 64 (75%) articles for which we were able to obtain a count had a median number of calibrated parameters of 10 (range 1 – 96). A histogram of the number of calibrated parameters is provided in figure B in S2 Appendix. We found that studies specifying a sampling strategy had higher numbers of calibrated parameters than those for which we could not identify the parameter search strategy (p<0.05, Kruskal-Wallis test followed by BH corrected Wilcoxon rank-sum tests, Fig 3).*

**Fig3. Boxplots of the number of target statistics by the parameter search strategy.**

*For 55 (66%) articles, we obtained counts for both the number of target statistics and the number of calibrated parameters. For several of these articles (17/55), the number of calibrated parameters appeared to exceed the number of calibrated parameters. A plot of the number of target statistics against the number of calibrated parameters is provided in figure C in S2 Appendix.*

*The size of the simulated population was explicitly mentioned in 54 (64%) of the 84 included articles, for another 9 (11%) articles it was possible to deduce this number from either text or figures. The remaining 21 (25%) articles either provided incomplete information (3/21) or no information at all (18/21). For the 63 (75%) articles for which we obtained a number, the median population size was 78000 (range: 250 - 47000000). A histogram of the log_10_ of the size of the simulated population is provided in figure D in S2 Appendix.”*

- Authors mention that ‘For several articles, the number of calibrated parameters appeared to exceed the number of target statistics.’ But do not specify how many.

***Response:*** *Please see the response to the reviewer’s previous comment. We added the following sentence to the results: “For many of these articles (17/55) the number of calibrated parameters appeared to exceed the number of calibrated parameters. A plot of the number of target statistics against the number of calibrated parameters is provided in figure C in S2 Appendix.”*

- Numbering format could be improved throughout the text.

***Response:*** *We improved the numbering format throughout the text. For example: ”Of the included articles, 40 (48%) combined a quantitative measure of GOF with an algorithmic parameter-search strategy, which was an optimisation algorithm (14/40) or a sampling algorithm (26/40) (see Figure 2). For the remaining 44 (52%) articles, the parameter-search strategy could either not be identified (32/44) or was described as an informal, non-reproducible method (12/44).”*

- Authors mention ‘The number of articles selected by publication year increased from seven in 2013 to 20 in 2018’. It might be interesting to look at the number of articles among these which reported calibration.

***Response:*** *We hope that we correctly understand the suggestion by the reviewer. Since all included articles reported to perform calibration, but not all articles reported on the calibration method, we inferred that the reviewer wanted to know whether reporting on or the type of parameter search strategy changed over time. We did not find any clear relationships between parameter search strategies and either publication year or pathogen. This information can be found in S1 Appendix. (See excerpt below). We added the following sentence to the manuscripts results section: “*Tables A, B and C in S1 appendix show that there is no convincing evidence that the parameter search strategy changed with publication year or differed by disease studied.”

**Table A. Parameter search strategy categories by year of publication**

|  | 2013 | 2014 | 2015 | 2016 | 2017 | 2018 |
| --- | --- | --- | --- | --- | --- | --- |
| Informal/Unidentifiable | 2 | 4 | 11 | 10 | 8 | 9 |
| Optimization | 2 | 2 | 5 | 0 | 1 | 4 |
| Sampling | 3 | 1 | 6 | 3 | 6 | 7 |

**Table B. Reporting on a parameter search strategy (y/n) by year of publication**

|  | 2013 | 2014 | 2015 | 2016 | 2017 | 2018 |
| --- | --- | --- | --- | --- | --- | --- |
| Not reported (unidentifiable onlyl) | 2 | 4 | 6 | 6 | 7 | 7 |
| Reported (informal, optimization & sampling) | 5 | 3 | 16 | 7 | 8 | 13 |

- Except this sentence ‘Only 32 (38%) articles mentioned that a validation of the model had been performed’ being used several times, one would not see any information about validation.

***Response:*** *We agree with the reviewer that we provide very little information on validation. Yet we think it is important to note the number of articles reporting to perform validation, as it potentially provides a check on calibration although the jury is still out on whether validation should be performed. We added a section to the part in the discussion on whether the target statistics provide enough information to calibrate the parameters: “Another related problem is that of validation, i.e. testing model performance on data that was not included in the calibration step. There is considerable debate on when data should be reserved for this purpose.”*

Discussion

- Can be read ambiguously ‘While algorithmic parameter-search strategies favour reproducibility, unclear or incomplete reporting, and non-disclosure of software code can still prevent reproducibility’

***Response:*** *We changed this sentence as follows: “While algorithmic parameter-search strategies are in principle reproducible, unclear or incomplete reporting, or non-disclosure of software code can render them de facto non-reproducible.”*

- Repetitive ‘These issues suggest that there is a need for research comparing the performance of calibration methods to inform decisions about the choice of parameter-search strategy and GOF’ and ‘Our review serves as a primer for methodological studies aimed at understanding the relative performance of alternative calibration methods.’

***Response:***  *We removed the latter sentence from the manuscript.*

- ‘Authors say ‘Manual adjustment of parameter values and visual inspection of GOF, on the other hand, does not favour reproducibility ... Nevertheless, such informal methods may perform equally ... and can be useful for simple didactic purposes.’ Would that mean manual adjustments do not favour reproducibility and yet one can use it? What should the readers infer from this?

***Response:*** We thank the reviewer for pointing out how these sentences are open to misinterpretation. *We changed the section to make it clear that we think these methods should not be used to inform public health: “Manual adjustment of parameter values and visual inspection of GOF, may perform equally well compared to other methods in terms of GOF alone, may provide researchers with valuable insights into and familiarity with the model, and can be useful for purely didactical purposes. However, we advise against using these methods in analyses intended to inform public health. As they do not favour reproducibility, involve subjective judgment, and usually lead to the acceptance of a single parameter set (i.e. does not provide parameter uncertainty).“*

**Reviewer #2:**

1. Line 98: In addition to the stochasticity, the number of parameters to estimate in IBM indicates the complexity of the parameter-search strategy (i.e. estimating only one parameter is the search on one-dimensional real space (1D), estimating two parameters is the search on two-dimensional real space (2D), and so on). The authors may comment in the introduction to show how the complexity of calibration varies between IBMs.

***Response:*** *We agree with the reviewer that this is an important fact and added the following sentences to the introduction:* ***“****IBMs vary in their complexity (i.e. the number of parameters) and the amount of data available for calibration and validation. Simulation-based calibration of IBMs of higher complexity is typically more computationally intensive.*

2. Line 144: It is better to provide a reference for the most investigated epidemics.

***Response:*** *We added a reference for the most investigated epidemics.*

3. Line 155: Is there any reasons why the authors did the search from 2013?

***Response:***  *We agree with the reviewer that performing the search from 2013 is arbitrary. The decision was based on our attempt at balancing the ability to complete the analysis within a reasonable time window with the limited resources we had, while still obtaining a comprehensive and informative overview of the literature. A time span of six years seemed to be long enough to yield a sizeable amount of information and to potentially observe any (recent) time trends, and short enough to be feasible and to speak to recent practices in model calibration in epidemiological modelling studies.*

4. Line 179: “the goal” need to be clarified along this line.

***Response:*** *We changed this line into: “For each article, we extracted information on the objective of the study (i.e. estimating the effect of an intervention, investigating a behavioural or biological explanation for the observed infectious disease outbreak or other goals including estimation of parameters or model development), the parameter-search strategy and the GOF measure, the rationale for choosing this calibration strategy over alternatives, and model validation.”*

5. Fig 2 lists the different methods of parameter search strategy found, but without minimal explanations. It is better to include in the appendix a table that explains these methods for non-modellers.

***Response:*** *We added such a table to the appendix as S2 Table, and refer to it in the text.*

6. Line 258: “…while the rest either relied on visual inspection as a GOF (14 articles) or used a quantitative GOF (five articles)...” The authors should discuss why some studies use manual fitting instead of formal parameter-search strategies. This manual fitting is impossible in case of multi-fitting parameters. Another issue of manual fitting is not reproducible due to the stochasticity in IBM.

***Response:*** *We thank the reviewer for this suggestion and included this suggestion in the discussion of the manuscript as follows: “On occasion, authors justified their choice of an informal method by indicating that algorithmic calibration methods did not converge to provide parameter estimates, or failed to provide a satisfactory fit to the targets. A potential explanation for non-convergence of an algorithmic calibration method is that the parameters in question are unidentifiable, which is the case when a vast array of different parameter combinations provide a comparably good fit to the target statistics. Performing manual calibration in this instance will deliver one set of parameter values out of all of the parameter combinations that provide a fit. However, using this single parameter combination hides the fact that there is not enough information to uniquely identify the best parameter values. Additionally, unless the seeds of random number generators are being controlled, model-stochasticity inherent to IBMs renders manual calibration non-reproducible. Furthermore, model-stochasticity provides the possibility that a great fit is found by chance for a parameter combination for which the probability of observing the target statistics is lower than for other parameter combinations.”*

7. Line 300: A reference along this line is very useful for the simulation tools.

***Response:*** *We added appropriate citations for the simulation tools.*

8. This systematic review provides interesting data about the methods of calibration of IBMs, but it did not provide a comparison, which method is best, or what is the strength and limitations of each method. The authors should clarify this point in the limitation section.

***Response:*** *We added the following sentence to the limitations section: ”**A final limitation is that we did not go into the strengths and weaknesses of each method. Existing literature compares the performance of alternative algorithms for calibrating the same model but does not allow us to draw general conclusions. As a starting point for comparison, we provide a brief description of calibration methods in S2 Table. ” Additionally, we incorporated the reviewer's comment in our suggestion for future research in the discussion: “**There is a need for simulation studies that compare the performance, strengths and limitations of different methods for calibrating IBMs to epidemiological data .”*

9. Minor comment: There is an issue in a cell in Table 1.

***Response:*** *Thank you for pointing this out, we formatted table1 and resolved the issue.*

**Reviewer #3:** The authors did a tedious and very valuable work in order to identify articles that used an individual-based model (IBM) to fit to data in HIV, tuberculosis (TB) and malaria, and assess the proportion of them that reported the parameter-search strategy and the type of parameter-search strategy used. This work is particularly important as one aims for more transparency on the optimization methods used in modelling works. However, the authors could go a little bit further in order to answer questions such as:

• Does the proportion of articles reporting the parameter search strategy used vary according to the disease studied (HIV, TB, malaria)?

***Response:*** *We addressed the question posed by the reviewer by making a table of the parameter search strategies per disease studied (See below). Additionally, we performed a statistical test and concluded that we could not see any clear relationship between reporting on calibration and of the pathogen studied (Statistical test provided in S1 Appendix). We added this information in S1 Appendix (See excerpt below). We added the following sentence to the results section of the manuscript: “The proportion of articles opting for a certain parameter search strategy did not vary according to the disease studied (i.e. HIV, TB, malaria) or the year of publication (i.e. 2013 - 2018), for details see S1 Appendix.”*

**Table A. Parameter search strategy by year of publication**

|  | 2013 | 2014 | 2015 | 2016 | 2017 | 2018 |
| --- | --- | --- | --- | --- | --- | --- |
| Informal/Unidentifiable | 2 | 4 | 11 | 10 | 8 | 9 |
| Optimization | 2 | 2 | 5 | 0 | 1 | 4 |
| Sampling | 3 | 1 | 6 | 3 | 6 | 7 |

**Table C. Parameter search strategy by disease studied (i.e. HIV, TB, malaria)**

|  | HIV | TB | Malaria |
| --- | --- | --- | --- |
| Informal/Unidentifiable | 33 | 3 | 8 |
| Optimization | 9 | 4 | 1 |
| Sampling | 16 | 3 | 7 |

• What about uncertainty? Is the uncertainty related to the parameter-search strategy (e.g. confidence interval or credibility interval) reported? Does the proportion of article reporting uncertainty depend on the field (i.e. HIV, TB, malaria) or on the parameter-search strategy used (sampling or optimization strategy)?

***Response:*** *We agree with the reviewer that uncertainty is an interesting aspect of model calibration. Two aspects of uncertainty can be discerned, the first is whether a confidence interval or credibility interval for the calibrated parameters was reported, the second is which sources of uncertainty are propagated in the model results. In the latter, we distinguish model stochasticity (i.e. random variation in model output, induced by running the model for the same parameter value with a different random seed) and parameter uncertainty (i.e. the extra uncertainty in model output induced by running the model for multiple draws of the posterior distribution of model parameters). The ability to propagate parameter uncertainty in the model results depends on the parameter search strategy. As we note in our manuscript: “Parameter distributions obtained from sampling algorithms allow for the representation of correlations between parameters and for parameter uncertainty to be incorporated into model projections” and “Optimisation algorithms provide only point estimates of parameters; once these are found, another algorithm may be used to obtain confidence intervals (e.g. the profile likelihood method, Fisher information, etc.).”. Therefore, only analyses using sampling-based calibration methods and optimisation methods using algorithms to obtain confidence intervals can incorporate parameter uncertainty in their model projections.*

• How many parameters are estimated in each study? Does this number depend on the parameter search strategy? Are the most complex models (i.e. the ones with the highest number of parameter to estimate) the ones that do not report uncertainty/search strategy?
Answering these questions could help better identify the articles that report searching method less frequently (e.g. assess whether it is related to the field studied). Reporting information about the number of parameters estimated and whether uncertainty is reported could also provide a wider understanding of the issue of lack of transparency that we face in some fields.

***Response:*** *We revised the analysis of the number of target statistics and the number of calibrated parameters. Please see the response to reviewer 1 on page 3.*

**In addition, I have a few minor comments:**
• Lines 49-54: This is mainly repetition of the results already presented in the previous paragraph. Consider removing this paragraph and replacing by a discussion of the results, e.g. something that looks like the second part of “Author summary” (lines 65-72).

***Response:*** *We removed the repetitive sentences from the abstract. The last part of the abstract, in which most of the repetition took place, now reads: “Reporting on calibration methods is far from optimal in epidemiological modelling studies of HIV, malaria and TB transmission dynamics. The adoption of better documented, algorithmic calibration methods could improve both reproducibility and the quality of inference in model-based epidemiology. There is a need for research comparing the performance of calibration methods to inform decisions about the parameter-search strategies and GOF measures.”*

• Line 98-99: To me, it is not clear why a greater complexity could make exact likelihood calculation impossible. I would rather say that greater complexity prevent from identifying the exact maximum likelihood estimator, but it should not prevent the model to calculate the exact likelihood.

***Response:*** *We thank the reviewer for bringing this to our attention; we agree that the word exact is used inadequately. Greater complexity often, but not always - as the reviewer notes - makes explicit likelihood calculation impossible. We changed the corresponding section of the text as follows: Parameter calibration is often difficult for IBMs because their greater complexity can render the likelihood function analytically intractable (i.e. it is impossible to write down the likelihood function in closed form) or prevents explicit numerical calculation of the likelihood function.” We continue to explain likelihood calculation in the next paragraph of the introduction where we added: “Quantitative measures of GOF include distance measures (e.g. relative distance, squared distance) and measures based on a surrogate likelihood function: the likelihood of observing the target statistic under the assumption that the model output is a random draw from a presumed distribution (e.g. binomial for prevalence statistics). As the model output is not necessarily distributed as presumed, we refer to this likelihood as the “surrogate” likelihood.”*

• Line 129: What kind of stochasticity do you mention here? Would it not be relevant to report stochasticity in your systematic review as well?

***Response:*** *We changed the sentence on line 129 to clarify the term stochasticity:*  ***“****The impact of stochasticity (i.e. random variation in model output, induced by running the model for the same parameter value with a different random seed) on the model results was summarized in nearly half (15/32) of the articles.” We acknowledge the reviewer's concern that it would be relevant to report on stochasticity in our systematic review but unfortunately did not collect this information for the current review.*

• Line 155: Why only from 2013 and not before?

***Response:***  *We agree with the reviewer that doing the search from 2013 is arbitrary, we had to choose to limit the number of articles we needed to sift and then review while still obtaining an overview of the literature. We added the following sentence to the methods section of the manuscript: “Six years seemed to be long enough to yield a sizeable amount of information and to potentially observe any (recent) time trends, and short enough to be feasible and to speak to recent practices in model calibration in epidemiological modelling studies.”*

• Line 166: Maybe you could mention before (in abstract?) that you will focus on studies informing public health policy.

***Response:*** *We agree with the reviewer and have incorporated this information in the abstract in the following sentence: “We identified articles on PubMed employing simulation-based methods to calibrate IBMs* ***informing public health policy*** *in HIV, tuberculosis, and malaria epidemiology that were published between 1 January 2013 and 31 December 2018. ”*

• Line 241: In the “GOF” column of Table 1, there is some formatting problem, as we can not read the whole text in the “Suen et al.” column.

***Response:*** *Thank you for pointing this out, we formatted Table 1 to resolve the issue*

• Line 271: It is not clear how you could obtain a weight (i.e. a number) with the ”inverse of the confidence interval” which are two numbers.

***Response:*** *We thank the reviewer for picking up this unclarity. We changed the concerning sentence as follows: “for example, by weighting based on the inverse of* ***the width of*** *the confidence interval around the data.“*

• Lines 309-323: Consider rewriting this paragraph, as, as it is now, it repeats what you already said in the “Results” section. I see the point of summarizing the results, but this could be in a more concise way.

***Response:*** *We changed the first part of the discussion by removing repetitions and making it more concise as follows: “We found that more than half of IBMs used non-reproducible or subjective calibration methods. Articles that reported the use of formal calibration methods used various parameter-search strategies and GOF measures. Only one-third of articles used calibration methods that allow for the quantification of parameter uncertainty. These findings are important because choices concerning the calibration method can have an important effect on model results and policy implications.”*

• Line 377: Was it not 48% (40/84) before, instead of 49%?

***Response:*** *Yes, we changed the respective percentage to 48%.*
