# Supplementary material for: Calibration of individual-based models to epidemiological data: A systematic review
Source: PLoS Comput Biol. 2020 May 11;16(5):e1007893. doi: 10.1371/journal.pcbi.1007893 (PMC7241852; doi:10.1371/journal.pcbi.1007893)
Supplement: Attachment — Submitted filename: Response_to_reviewers.docx [file pcbi.1007893.s010.docx]

**Comments to the Authors:
Please note here if the review is uploaded as an attachment.**

Reviewer #1: Good job. I have no additional comments.

Reviewer #2: The authors have satisfactorily responded to all my questions and made the necessary changes to the manuscript.

Reviewer #3: All the previously reported issues have been addressed by the authors. The authors might however check the following issue. In Fig3, title (and legend) mentioned that the figure reports the number of target statistics, while the y-axis label mentions the number of calibrated parameters. This must be corrected. Additionally, the authors could present both figures (number of targets and number of calibrated parameters according to the parameter search strategy) side by side in the main manuscript.

***Response:*** *We thank the reviewer for pointing out the issue with Fig3, and we changed the figure according to the recommendations provided.*

*In addition, we made some minor edits in the manuscript, and we provide a highlighted version indicating the changes made.*
